# Understanding Bridging Sites and Accelerating Quantum Efficiency for Photocatalytic CO_2_ Reduction

**DOI:** 10.1007/s40820-023-01221-3

**Published:** 2023-11-06

**Authors:** Kangwang Wang, Zhuofeng Hu, Peifeng Yu, Alina M. Balu, Kuan Li, Longfu Li, Lingyong Zeng, Chao Zhang, Rafael Luque, Kai Yan, Huixia Luo

**Affiliations:** 1 School of Materials Science and Engineering, State Key Laboratory of Optoelectronic Materials and Technologies, Guangdong Provincial Key Laboratory of Magnetoelectric Physics and Devices, Key Lab of Polymer Composite and Functional Materials, Sun Yat-Sen University, No. 135, Xingang Xi Road, Guangzhou, 510275 People’s Republic of China; 2 School of Environmental Science and Engineering, Sun Yat-Sen University, No. 135, Xingang Xi Road, Guangzhou, 510275 People’s Republic of China; 3 Departamento de Química Orgánica, Universidad de Córdoba, Campus Universitario de Rabanales, Edificio Marie Curie (C3), 14014 Córdoba, Spain; 4 Center for Refining and Advanced Chemicals, King Fahd University of Petroleum and Minerals, 31261 Dhahran, Saudi Arabia; 5 Universidad ECOTEC, Km 13.5 Samborondón, EC092302 Samborondón, Ecuador

**Keywords:** Quantum efficiency, Electronic structure, Steric interaction, Bridging sites, CO_2_ reduction

## Abstract

We report a novel double-shelled nanoboxes photocatalyst architecture with tailored interfaces that accelerate quantum efficiency for photocatalytic CO_2_ reduction reaction (CO_2_RR) via Mo**–**S bridging bonds sites in S_v_–In_2_S_3_@2H–MoTe_2_. The X-ray absorption near-edge structure shows that the formation of S_v_–In_2_S_3_@2H–MoTe_2_ adjusts the coordination environment via interface engineering and forms Mo**–**S polarized sites at the interface. The interfacial dynamics and catalytic behavior are clearly revealed by ultrafast femtosecond transient absorption, time-resolved, and in situ diffuse reflectance–Infrared Fourier transform spectroscopy. A tunable electronic structure through steric interaction of Mo**–**S bridging bonds induces a 1.7-fold enhancement in S_v_–In_2_S_3_@2H–MoTe_2_(5) photogenerated carrier concentration relative to pristine S_v_–In_2_S_3_. Benefiting from lower carrier transport activation energy, an internal quantum efficiency of 94.01% at 380 nm was used for photocatalytic CO_2_RR. This study proposes a new strategy to design photocatalyst through bridging sites to adjust the selectivity of photocatalytic CO_2_RR.

**Supplementary Information:**

The online version contains supplementary material available at 10.1007/s40820-023-01221-3.

## Highlights

- The S-vacancies result in the change of d-band electronic state of Mo.
- An internal quantum efficiency of 94.01% at 380 nm for photocatalytic CO_2_ reduction reaction (CO_2_RR).
- The Mo–S bridging bonds optimize adsorption energies and accelerate CO_2_RR kinetics.

**Supplementary Information:**

The online version contains supplementary material available at 10.1007/s40820-023-01221-3.

## Graphical abstract

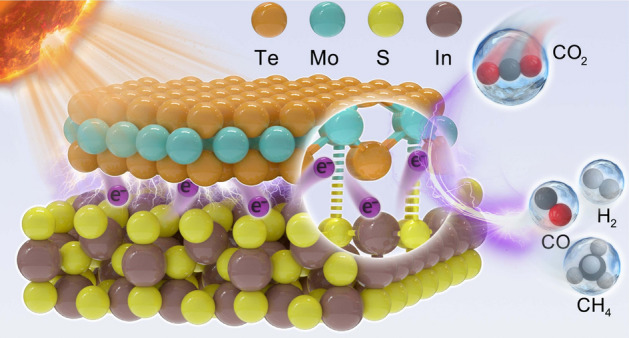

## Introduction

Two of the largest research challenges confronting the world today are the ever-rising need for clean energy and the global crisis of climate change [1]. Converting solar energy by means of the mild light-driven chemical reactions is of far-reaching importance for the development of green and sustainable energy sources [2]. A wide variety of exciting products resulting from carbon dioxide (CO_2_) conversion are CH_3_OH, CH_4_, and other available organic compounds, which are stable, nontoxic substances with considerable market potential in various applications [3, 4]. Unfortunately, CO_2_ reduction reaction (CO_2_RR) frequently is difficult to carry out owing to CO_2_ being thermodynamically stable, resulting in extraordinarily andante reaction kinetics during the photoreduction process. Beyond that, the conversion of CO_2_ molecules competes with other side reactions, such as the common hydrogen (H_2_) evolution reaction (HER, 2H^+^ + 2e = H_2_), which memorably declines the production of reduced carbon products. For this reason, highly selective, stable and efficient catalysts are required to facilitate photocatalytic CO_2_RR, overcoming significant energy barriers and tuning the reaction pathways to the formation of CH_3_OH and CH_4_, as well CO [5].

Two-dimensional (2D) materials, especially transition metal dichalcogenides (TMDs, MX_2_) [M refers to a transition metal (Ta, Nb, Mo, and W, etc.), and X denotes as a chalcogen (S, Se, and Te, etc.)] have been extensively studied and applied in many fields for decades, because of their low cost, superior electronic, topological and mechanical properties, as well as their ultrathin low-dimensional nature. By now, the members of TMDs group, on account of their unique electronic and atomic structural behavior, have been widely considered to be an ideal alternative for photocatalytic and electrocatalytic CO_2_RR. Despite the emergence of many fascinating preponderances, inadequate intrinsic electrical transport and inactive substrate surfaces in the TMDs group severely impede their application in photocatalytic and electrocatalytic CO_2_RR, for instance, typical molybdenum sulfide (MoS_2_) and molybdenum selenide (MoSe_2_) materials [6]. It is interesting to notice that molybdenum telluride (MoTe_2_) has recently attracted attention due to its metallic conductivity and outstanding electron transport capacity. The existence of different phases of MoTe_2_ provides the feasibility of developing a wealth of novel structures and gadgets, including a semiconducting 2H-phase (prismatic trigonal structure; bandgap of about 1.0 eV), a topological semimetallic 1 T-phase (twisted octahedron; energy gap covered near the Fermi energy level (*E*_F_)), and a possible topological superconducting *T*_d_-phase, which facilitates the commercial application of MoTe_2_ in the physical industry [7, 8]. Despite this, the construction of catalytically active nano-heterojunctions with novel compositions and microscopic morphologies remains a major challenge requiring urgent breakthroughs due to the challenges of synthetic methods and design ideas.

**Scheme 1:**
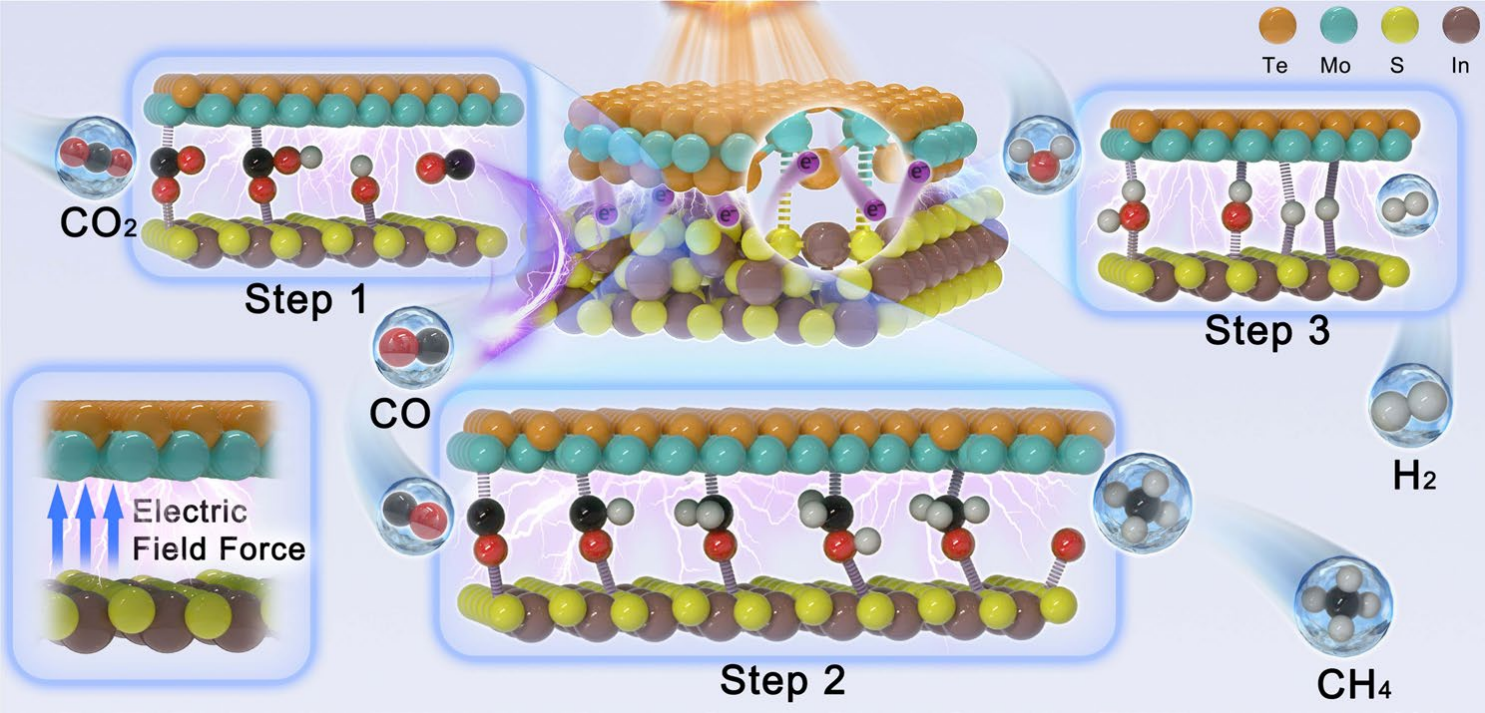
Schematic illustration for modulating Mo**–**S bonds coupling step in CO_2_ reduction pathways over S_v_–In_2_S_3_@2H–MoTe_2_(5)

Based on previously reported studies, designing double-shelled hollow structures of semiconductor nanomaterials is one of the most effective stratagems to for improving light utilization, tuning electronic structure and steric interaction of chemical bonds, accelerating interfacial contact, providing more catalytic reaction sites and promoting effective carrier separation and transfer. Given this, we strategically proposed a “double-shelled nanoboxes” design for S_v_–In_2_S_3_@2H–MoTe_2_ catalysts, in which 2H–MoTe_2_ was coated on S_v_–In_2_S_3_ single-shelled nanoboxes to form the Mo**–**S bonds of S-vacancies-rich junction structure. Assisted by the robust built–In electric field (IEF) and Mo–S bridging bonds of S_v_–In_2_S_3_@2H–MoTe_2_(5), “S”-scheme charge separation is notably facilitated, resulting in an internal quantum efficiency (IQE) calculated via photocatalytic CO_2_RR of 94.01% (IQE_cr_) at 380 nm. Particularly, the Mo–S bridging bonds can reduce the adsorption energy barriers of *OCHO and *CHO species and effectively regulate the formation energy barriers of CO, H_2_, and CH_4_, thus enhancing the photocatalytic activity (Scheme 1).

## Experimental Section

### Materials

Indium chloride (InCl_3_) and sodium hydroxide (NaOH) were purchased from Sinopharm Group Chemical Reagent Co., Ltd. Copper sulfate (CuSO_4_∙5H_2_O) was purchased from Acros Organics with a purity of over 99.99%. Tellurium powder (Te, more than 200 mesh) was purchased from Shanghai Aladdin Biochemical Technology Co., Ltd. Hydrazine hydrate (N_2_H_4_·H_2_O) was purchased from Sinopharm Group Chemical Reagent Co., Ltd. Sodium molybdate (Na_2_MoO_4_·2H_2_O) was supplied by Aladdin Reagent Co., Ltd. (Shanghai, China). Polyvinylpyrrolidone (PVP, Mw = 400,000) was provided by Shanghai Ryon Biotechnology Co., Ltd. (Shanghai, China). Sodium thiosulfate (Na_2_S_2_O_3_), with a purity of over 99.5%, was purchased from Shanghai Aladdin Biochemical Technology Co., Ltd. (Shanghai, China). Dimethyl sulfoxide (DMSO) and thioacetamide (C_2_H_5_NS) were purchased from Sigma with a purity of over 99.99%. All of the reagents used in our experiments were analytical purity and used without further purification.

### Preparation of In_2_S_3_, S_v_–In_2_S_3_, 2H–MoTe_2_ and S_v_–In_2_S_3_@2H–MoTe_2_

#### Preparations of the In_2_S_3_ SSNBs

The as-prepared In(OH)_3_ single-shelled nanoboxes (SSNBs) and 40 mg of C_2_H_5_NS were dispersed into 20 mL of ethanol. The solution was transferred into a Teflon-lined stainless autoclave and heated at 90 °C in an oven for 2 h. The precipitate was harvested by centrifugation, washed with DW and ethanol several times, and subsequently annealed in N_2_ at 300 °C for 2 h to obtain the In_2_S_3_ SSNBs.

#### Preparations of the S_v_–In_2_S_3_ SSNBs

S-vacancies-rich In_2_S_3_ SSNBs (S_v_–In_2_S_3_ SSNBs) were prepared via an N_2_H_4_·H_2_O-assisted hydrothermal method. Typically, the as-synthesized In_2_S_3_ SSNBs (100 mg) were dispersed into 20 mL of deionized water for 1 h, and 5 mL N_2_H_4_·H_2_O was added into the mixing solution and stirred for another 30 min. Afterward, the mixture was transferred to a 50 mL autoclave and maintained at a 240 °C oven for 5 h. Finally, the precipitate was separated by centrifugation, washed with DW and ethanol several times, then dried at 60 °C for 10 h.

#### S_v_–In_2_S_3_@2H–MoTe_2_ Double-Shelled Nanoboxes (DSNBs)

The S_v_**–**In_2_S_3_@2H**–**MoTe_2_ DSNBs were synthesized by a similar process to S_v_**–**In_2_S_3_ SSNBs, except that Na_2_MoO_4_·2H_2_O and Te powders were added to the mixture. The S_v_**–**In_2_S_3_@2H**–**MoTe_2_ DSNBs with a different mass ratio of 2H–MoTe_2_ to S_v_**–**In_2_S_3_ (1.0%, 3.0%, 5.0%, 7.0%, and 9.0%) were synthesized by adjusting the addition of Na_2_MoO_4_·2H_2_O and Te, and the synthesized samples were labeled as S_v_–In_2_S_3_@2H–MoTe_2_(1), S_v_–In_2_S_3_@2H–MoTe_2_(3), S_v_–In_2_S_3_@2H–MoTe_2_(5), S_v_–In_2_S_3_@2H–MoTe_2_(7), and S_v_–In_2_S_3_@2H–MoTe_2_(9), respectively. For comparison, the pure 2H–MoTe_2_ nanosheets (NSs) were prepared following the above steps without adding S_v_–In_2_S_3_ SSNBs.

### Characterization

The crystal phase properties and morphologies for various samples were analyzed with an X-ray diffraction (XRD) and scanning electron microscopic (SEM) images, respectively. To further determine the morphology and crystal lattice structure of the materials, transmission electron microscopy (TEM) images were taken using a Hitachi H-7650 transmission electron microscope at an acceleration voltage of 100 kV. High-resolution TEM (HRTEM), high-angle annular dark-field scanning transmission electron microscopy (HAADF-STEM), and energy-dispersive X-ray spectroscope (EDX) mapping were carried out on a JEOL ARM-200F field-emission transmission electron microscope operating at 200 kV accelerating voltage. X-ray photoelectron spectroscopy (XPS, ESCALAB250) of Thermo was used to study the element combination and valence of the materials in this work. Temperature programmed desorption (TPD) profiles of the samples were recorded by a Micromeritics AutoChem II 2920 chemisorption analyzer with a thermal conductivity detector (TCD). The Brunauer–Emmett–Teller (BET) specific surface areas and porosity of the samples were determined using micromeritics (ASAP 2460 U.S.A.) surface area and porosity analyzer. The ultraviolet–visible diffuse reflectance spectra (DRS) were recorded using a UV–vis instrument (Japan). Steady-state photoluminescence (PL) and time-resolved photoluminescence (TRPL) spectra were collected on the F-7000 fluorescence spectrophotometer (Japan, Hitachi, *λ*_ex_ = 319 nm, *λ*_em_ = 610 nm) and FLS920 fluorescence lifetime spectrophotometer (Edinburgh Instruments, UK), respectively.

### Photocatalytic CO_2_RR Experiment

The photocatalytic CO_2_RR measurement was conducted by the Lab Solar-Ш AG system (Perfect light Limited, Beijing). A 300 W Xe lamp equipped with a UV cut-off filter (λ > 400 nm) was adopted as the light source, calibrated by a CEL-NP2000 Optical Power Meter (Beijing China Education Au-light Co., Ltd.). The intensity of visible-light was 360 mW cm^−2^. The instrument was initially vacuum-treated 3 times and then pumped with high-purity CO_2_ to reach atmospheric pressure. 50 mL of KHCO_3_ (0.5 M) was injected into this setup for further photocatalysis. Gas products were detected using a Barrier Discharge Ionization Detector (BID) detector with gas chromatography (Shimadzu, Nexis GC-2030). ^1^H nuclear magnetic resonance (NMR) spectra were recorded on a Bruker DPX 400 spectrometer to detect the liquid products.

The specific operation for cycling experiments: at first, the cycling stability test was measured by repeating the above operations with a 20 mg sample every 5 h. During the light irradiation, the carbon-based gas products were qualitatively analyzed by Agilent GC7890B gas chromatograph by identifying the chromatographic peaks. Other gas products, such as O_2_, were analyzed by a thermal conductivity detector. The carrier gas was Ar with a flow rate of 20 mL min^−1^, and the column temperature was 393 K. All gas products were injected by an automatic online sampler with 1.0 mL gas. After the reaction, the liquid products were quantified by NMR spectroscopy, in which DMSO solution was used as the internal standard. The temperatures of the solutions were controlled at 298 ± 0.2 K by a recirculating cooling water system during visible-light irradiation [9].

### Computational Details

First-principles computations based on the density functional theory (DFT) were implemented in the Vienna Ab initio simulation package (VASP) [10]. The generalized gradient approximation (GGA) involving Perdew, Burke, and Ernzerhof (PBE) was used for calculating the exchange–correlation energy [11]. A 400 eV of cut-off energy was adopted for the plane-wave basis set in conjunction with the projector augmented wave (PAW) [12]. The energy and force convergence were set to be 1 × 10^−4^ and 5 × 10^−2^ eV, respectively. Here, a vacuum layer of 12 Å is chosen in the *z* direction to avoid interactions between periodically repeated slabs. The Brillouin zone was sampled using the Monkhorst–Pack scheme, K-points were generated by VASPkit [13], and the recommended value is 0.04 (2*π* × 0.04 Å^−1^). The van der Waals interaction was considered by using the DFT-D3 method [14].

The Gibbs free energy of the intermediates for HER and CO_2_RR process, that is, *CO_2_, *OCHO, OH*, CO*, CHO*, CH_2_O*, CH_3_O*, and *O, can be calculated as follows:

1 $$\Delta G \, = \, \Delta E_{{{\text{DFT}}}} + \, \Delta E_{{{\text{ZPE}}}} - \, T\Delta S$$

2 $$\Delta E_{{{\text{ZPE}}}} \, = \, \Delta \sum\nolimits_{i} {1{/}2 \, hv_{i} } \,$$

3 $$\theta_{i} \, = \, hv_{i} {\text{/k}}$$

4 $$S \, = \, \sum\nolimits_{{\text{i}}} {R[\ln (1 - e^{{ - \Theta_{i} /T}} )^{ - 1} + \Theta_{i} {/}T(e^{{\Theta_{i} /T}} - 1)^{ - 1} ]} \,$$

where Δ*E*_DFT_, Δ*E*_ZPE_, and Δ*S* are the total energy change, zero-point energy change, and the entropy change (Δ*S*) of each adsorbed state were calculated according to the standard molar Gibbs energy of formation at 298.15 K. *S* is the entropy, *h* is the Planck constant, *ν* is the computed vibrational frequencies, *Θ* is the characteristic temperature of vibration, *k* is the Boltzmann constant, and *R* is the molar gas constant. *T* is the temperature and is taken as 298.15 K. The entropy of other adsorbed states (*T*Δ*S*) is calculated from the vibrational frequencies associated with the standard modes in the harmonic approximation [15]. The contributions are listed (Tables S8 and S9). For adsorbates, *E*_ZPE_ and *S* are obtained from vibrational frequency calculations with harmonic approximation, and contributions from the slabs are neglected. In contrast, for molecules, these values are taken from NIST-JANAF thermochemical Tables [16].

## Result and Discussion

### Structural Characterization

The experimental sections and preparation procedure were provided in Supporting Information and Figs. S1–S3, including the synthesis of hollow In_2_S_3_ using Cu_2_O NCs as a template and the subsequent hydrothermal synthesis S_v_–In_2_S_3_ and 2H–MoTe_2_. With regard to the electron paramagnetic resonance (EPR) spectrum, S_v_–In_2_S_3_, and S_v_–In_2_S_3_@2H–MoTe_2_(5) represent a clear signal at *g* = 2.076 (Fig. S4a), further reconfirming that S_v_–In_2_S_3_@2H–MoTe_2_(5) and S_v_–In_2_S_3_ are rich in S-vacancies species [2, 17]. Of great importance, the higher concentration of S-vacancies species in S_v_–In_2_S_3_@2H–MoTe_2_(5) leads to significant changes in its electronic structure, steric interaction of chemical bonds and energy density distribution, thereby significantly enhancing its electrical conductivity, which is more beneficial for electrons transfer during photocatalytic CO_2_RR progress [18, 19]. As a comparison, there is no obvious S-vacancies signal of In_2_S_3_ in EPR spectrum. The XRD patterns of S_v_–In_2_S_3_ and In_2_S_3_ are well indexed to the tetragonal phase of In_2_S_3_ (JCPDS No. 73-1366), indicating their high purity without any other crystal structure changes (Fig. 2a). Interestingly, S_v_–In_2_S_3_ denote almost the same diffraction signals as the In_2_S_3_, and there are no other heterogeneous phases, which accounts for that the S-vacancies hardly affects original crystal phase structure of In_2_S_3_ [20]. Figure S5 exhibits that S_v_–In_2_S_3_@2H–MoTe_2_(5) possesses a micro/mesoporous structure with the size at range of 0–250 nm, and the average pore diameter is about 4.51 nm (Table S3). Furtherly, S_v_–In_2_S_3_@2H–MoTe_2_(5) has increased surface area that helps to increase contact with the reactants and shorten transmission path of the charge carrier [21]. According to above analysis, S_v_–In_2_S_3_@2H–MoTe_2_(5) has abundant catalytic active sites and porous structures, which is conducive to increase contact area with reactants, and facilitate escape of CH_4_, CO, and H_2_, thus improving photocatalytic CO_2_RR activity.

**Fig. 1:**
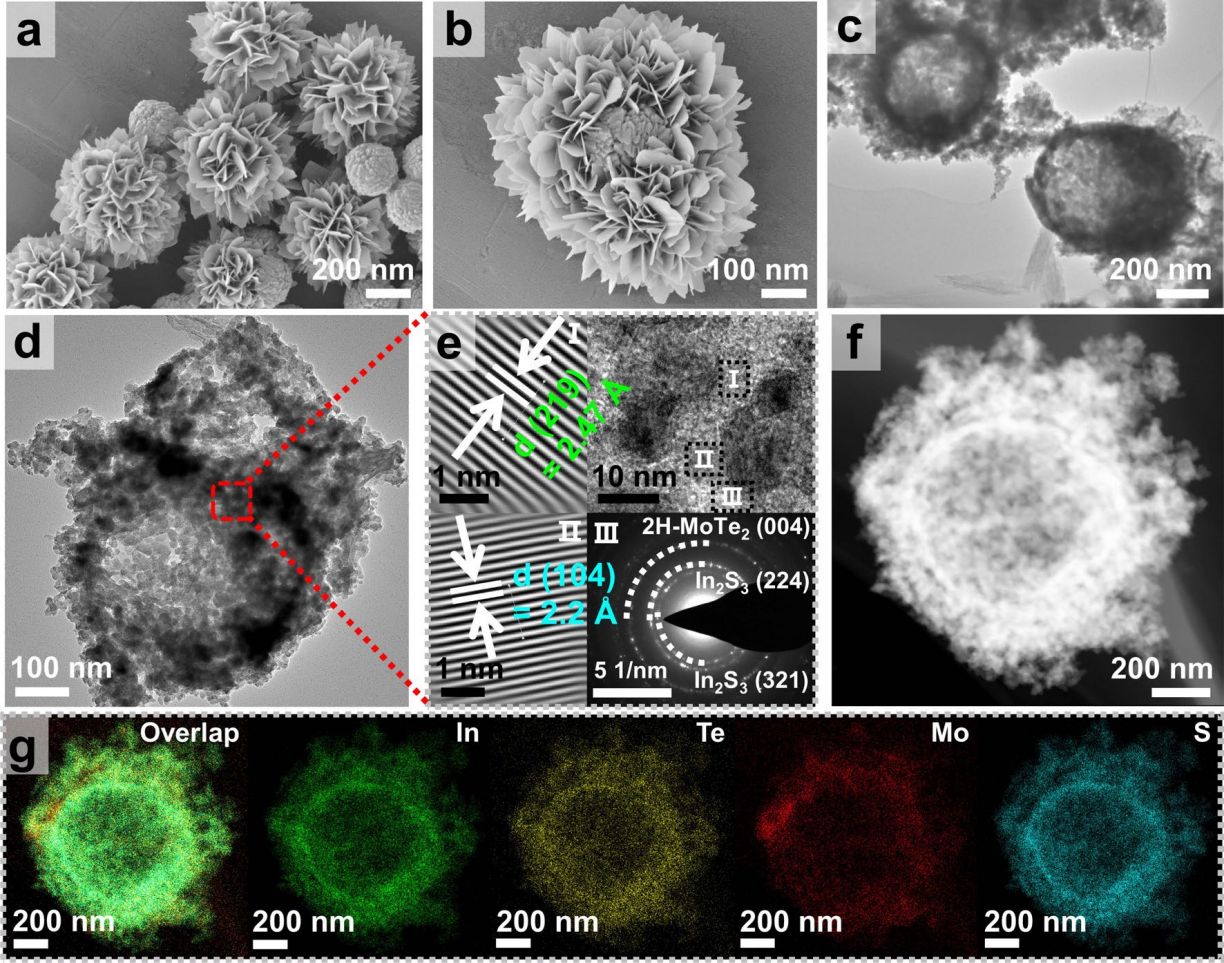
**a, b** SEM, **c, d** TEM,** e** HRTEM images, IFFT (top) and SAED patterns,** f** HAADF STEM, and **g** HAADF-STEM-EDS elemental mapping images of S_v_–In_2_S_3_@2H–MoTe_2_(5), respectively

The morphological characteristics of S_v_–In_2_S_3_@2H–MoTe_2_(5), S_v_–In_2_S_3_, In_2_S_3_, and 2H–MoTe_2_ were elaborately analyzed via SEM, TEM, HRTEM, HAADF-STEM, and HAADF-STEM-EDX elemental mapping images, respectively. As depicted in Fig. 1, the basic morphology of S_v_–In_2_S_3_@2H–MoTe_2_(5) is a double-shelled nanoboxes composed of a large number of ultrathin nanosheets on the exterior, which facilitates the exposure of active surface and the scattering phenomenon (Mie scattering) [22]. The EDX elemental mapping images of S_v_–In_2_S_3_@2H–MoTe_2_(5) present that Mo and Te elements are obviously distributed on the outer surface of S_v_–In_2_S_3_ (Fig. 1g), illustrating the successful formation of homogeneous nano-heterojunction structures between S_v_–In_2_S_3_ and 2H–MoTe_2_, which can be further proved that 2H–MoTe_2_ was directly grown and attached to S_v_–In_2_S_3_. Besides, it can be clearly seen that atomic data ratio of In/S and Mo/Te is about 1.00/1.48 and 1.00/2.05 (Table S1), which is extremely close to stoichiometric ratio in molecular formula of In_2_S_3_ and 2H–MoTe_2_. Furtherly, HRTEM images, inverse fast Fourier transform (IFFT) patterns, and lattice fringe profile (LFP) manifest distinctly visible lattice fringes with spacing of 2.47 and 2.20 Å (Fig. 1e), pointing precisely to (219) crystal plane of In_2_S_3_ (JCPDS:73-1366) and (104) crystal plane of 2H–MoTe_2_ (JCPDS:73-1650), respectively. The selected-area-electron-diffraction (SAED) pattern of S_v_–In_2_S_3_@2H–MoTe_2_(5) reveals a ring-like pattern of S_v_–In_2_S_3_ and 2H–MoTe_2_, confirming the presence of S-vacancies and polycrystalline features of composites.

### Electronic Structure Analysis

**Fig. 2:**
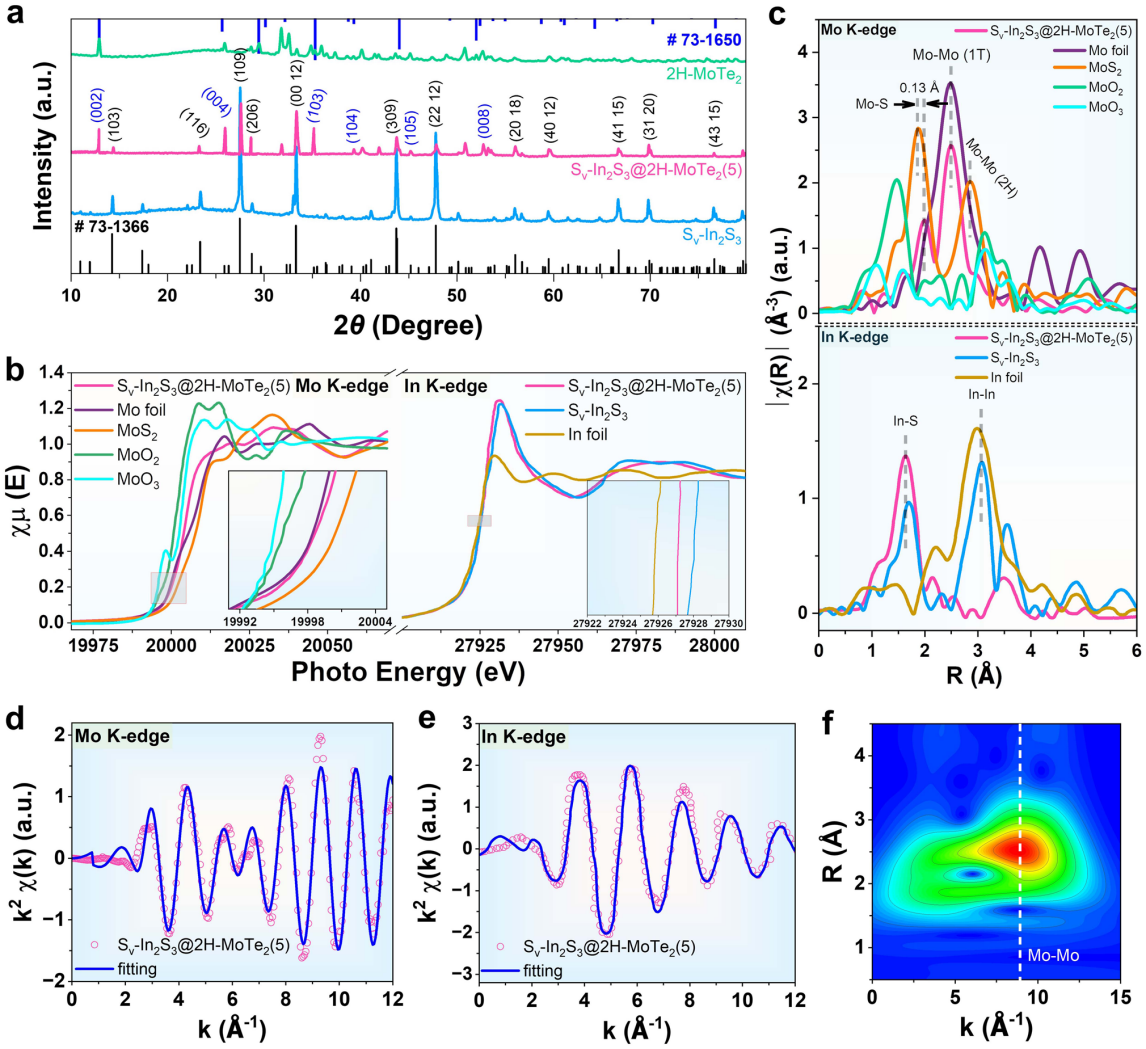
**a** XRD patterns of 2H–MoTe_2_, In_2_S_3_, S_v_–In_2_S_3_, and S_v_–In_2_S_3_@2H–MoTe_2_(5), respectively. **b** Comparison of Mo and In K-edge XANES spectra. **c** k^3^-weighted FT-EXAFS spectra of Mo and In at R space. **d** Mo K-edge EXAFS and **e** In K-edge EXAFS for S_v_–In_2_S_3_@2H–MoTe_2_(5), shown in k^2^ weighted k-space. **f** WT for k^3^ weighted EXAFS contour plots of S_v_–In_2_S_3_@2H–MoTe_2_(5)

The chemical composition and atomic state information of samples were compared by XPS. The complete scanning spectrogram is shown in Fig. S4b–f, and the specific atomic ratio is displayed in Table S2. In the Mo 3*d* core-level spectrum in Fig. S4e, the peaks of 3*d*_5/2_ and 3*d*_3/2_ appear on 225.7 and 229.0 eV, belonging to Mo–S bridging bonds in S_v_–In_2_S_3_@2H–MoTe_2_(5) [23]. The binding energies (B. E.) of Mo^4+^ at 227.9 and 231.1 eV in 2H–MoTe_2_ negatively shift to 225.7 and 228.9 eV in S_v_–In_2_S_3_@2H–MoTe_2_(5). It is speculated that the uniform distribution of S-vacancies in S_v_–In_2_S_3_ regulates the coordination environment of 2H–MoTe_2_, leading to a change in d-band electronic state of Mo [24]. The peaks at 159.1 and 161.6 eV in S 2*p* spectrum (Fig. S4f) represent S 2*p*_3/2_ and S 2*p*_1/2_ binding energies of S^2–^ in S_v_–In_2_S_3_@2H–MoTe_2_(5). The slight positive shift of S 2*p* peak (by 1.47 eV) is also attributed to the interfacial charge transfer of S_v_–In_2_S_3_ and 2H–MoTe_2_ [25, 26]. The negative shift of S 2*p* and positive shift of Mo^4+^ indicate the charge transfer from 2H–MoTe_2_ to S_v_–In_2_S_3_ at S_v_–In_2_S_3_@2H–MoTe_2_(5) (Fig. S4c). The X-ray absorption fine structure spectroscopy (XAFS) of Mo K-edge and In K-edge was employed to provide in-depth insights into atomic and electronic structure between S_v_–In_2_S_3_ and 2H–MoTe_2_ in S_v_–In_2_S_3_@2H–MoTe_2_(5). Figure 2b reveals the X-ray absorption near-edge structure (XANES) spectrum at Mo K-edge of samples along with Mo metal foil and MoS_2_ standard. In Mo and MoS_2_, Mo exit in 0 and + 4 oxidation states, respectively [27, 28]. The absorption edge of samples lies between that of Mo metal foil and MoS_2_. The Mo K-edge XANES spectrum of S_v_–In_2_S_3_@2H–MoTe_2_(5) demonstrates a negative shift in contrast with that of MoS_2_. The Mo valence of Mo–S bonds in S_v_–In_2_S_3_@2H–MoTe_2_(5) discloses a slight increase, corresponding with XPS results. The In K-edge XANES spectra of S_v_–In_2_S_3_@2H–MoTe_2_(5) and S_v_–In_2_S_3_, showed the + 3 valence state of In element in S_v_–In_2_S_3_@2H–MoTe_2_(5) and S_v_–In_2_S_3_ (Fig. 2b). The In EXAFS spectra shows the dominant peaks at 1.67 and 1.77 Å, corresponding to In** − **S and In**–**In coordination (Fig. 2c), respectively, which is consistent with XPS results (Fig. S12c). As observed in Figs. 2d, f and S6a, b, the extended X-ray absorption fine structure (EXAFS) spectrum for Mo sites shows two prominent peaks contributed by Mo–S and Mo**–**Mo bonds at 1.99 and 2.85 Å, respectively, implying the existence of Mo–S bridging bonds in S_v_–In_2_S_3_@2H–MoTe_2_(5) [29, 30]. Furthermore, the Mo K-edge EXAFS spectrum of S_v_–In_2_S_3_@2H–MoTe_2_(5) demonstrates a positive radial distance shift (0.13 Å) of Mo–S bonds compared to MoS_2_, further confirming the existence of Mo–S bridging bonds [31, 32]. In 2D color patch image obtained after EXAFS signal via wavelet transformation (WT), a high energy signal (red area) appears at 8.5 Å for S_v_–In_2_S_3_@2H–MoTe_2_(5) (Fig. 2f) and MoS_2_ (Fig. S6c), which corresponds to signal of Mo**–**Mo coordination bond [24, 33].

The difference in charge density between S_v_–In_2_S_3_ and 2H–MoTe_2_ can visually reflect the carrier transfer. In Fig. 4h and Fig. S14d, 3D charge density difference of S_v_–In_2_S_3_@2H–MoTe_2_(5) presented the existence of interfacial charge transfer between S_v_–In_2_S_3_ and 2H–MoTe_2_. It can be found from illustration that, the carriers are spontaneously transferred from S_v_–In_2_S_3_ to 2H–MoTe_2_ through the boundary, whereas holes gather on the side of S_v_–In_2_S_3_ in S_v_–In_2_S_3_@2H–MoTe_2_(5). Consequently, the charge accumulation occurred on 2H–MoTe_2_, whereas charge loss was observed on S_v_–In_2_S_3_ [34]. Finally, a strong built–In electric field (IEF) of Mo–S bonds from S_v_–In_2_S_3_ to 2H–MoTe_2_ is established on account of the redistribution of electrons after the contact between S_v_–In_2_S_3_ and 2H–MoTe_2_ [35]. The interfacial electrostatic interaction allows the electrons in conduction band (CB) of 2H–MoTe_2_ to recombine with the holes in valence band (VB) of S_v_–In_2_S_3_ through Mo–S bridging bonds, resulting in effective retention of electrons in CB of S_v_–In_2_S_3_.

### Photocatalytic CO_2_RR Performance

**Fig. 3:**
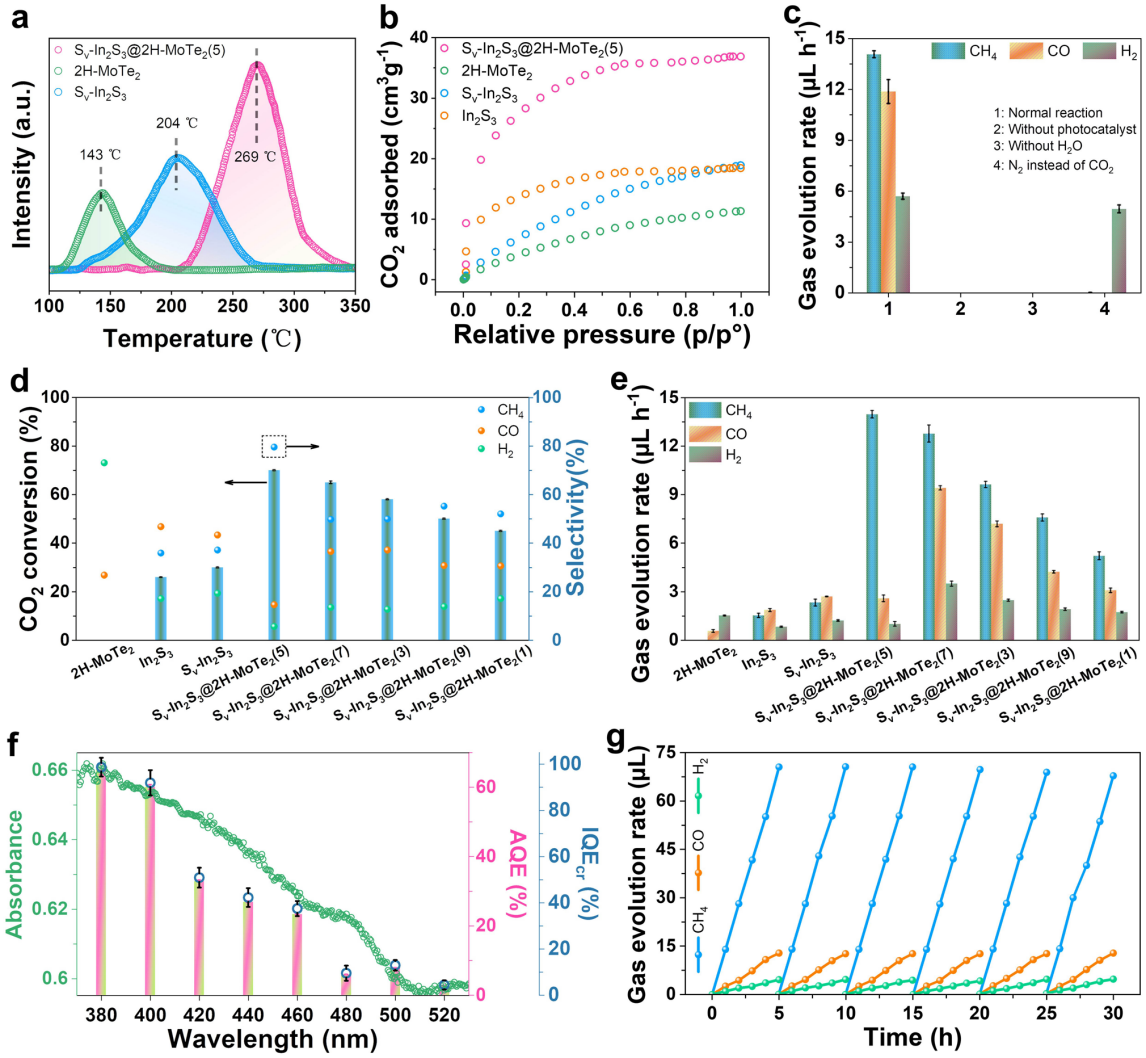
**a** CO_2_-TPD spectra of S_v_–In_2_S_3_, 2H–MoTe_2_, and S_v_–In_2_S_3_@2H–MoTe_2_(5), respectively.** b** CO_2_ adsorption isotherms of samples at 298 K. **c** Control experiments in several conditions. **d** CO_2_ conversion and product selectivity. **e** Yields of CO, CH_4_, and H_2_ for photocatalysts (KHCO_3_ solution). **f** AQE, IQE_cr_, and absorption spectrum of S_v_–In_2_S_3_@2H–MoTe_2_(5). **g** Stability test of with S_v_–In_2_S_3_@2H–MoTe_2_(5) in 6 cycles, where each photocatalytic cycle lasted for 5 h (KHCO_3_ solution). All the experiments were repeated at least 3 times in parallel to obtain an average value

CO_2_-TPD experiments were carried out on the photocatalyst to further explore the adsorption of CO_2_. Figure 3a presents CO_2_-TPD profiles of 2H–MoTe_2_, S_v_–In_2_S_3_, and S_v_–In_2_S_3_@2H–MoTe_2_(5), revealing the presence of prominent peaks in investigated temperature range and thus suggesting moderately primary CO_2_ adsorption centers on the surface of photocatalyst. The hollow porous nature of S_v_–In_2_S_3_@2H–MoTe_2_(5) (Fig. S5) enables high CO_2_ capture capability (36.83 cm^3^ g^−1^, Fig. 3b), facilitating CO_2_RR on S_v_–In_2_S_3_@2H–MoTe_2_(5). Herein, the higher CO_2_ adsorption capacity of S_v_–In_2_S_3_@2H–MoTe_2_(5) is due to its chemisorption for CO_2_ through the stronger coordination interaction of CO_2_ with Mo (+ 4) (Scheme 1, Mo–S bridging bonds). The photocatalytic CO_2_RR activity was evaluated via analyzing raw material and gas products produced at gas–solid interface in the absence of cocatalysts and photosensitizers (Fig. S7). The main products were examined by gas chromatography–mass spectra (GC–MS). During photocatalytic CO_2_RR process, the principal reduction products in system are CO, CH_4_, and H_2_, which is consistent with the previous reports in similar scenarios. The S_v_–In_2_S_3_@2H–MoTe_2_(5), In_2_S_3_, and S_v_–In_2_S_3_ show relatively lower photocatalytic CO_2_RR activity, with CH_4_-evolution rates of 13.97, 1.53, and 2.32 μL h^−1^, respectively (Figs. 3d, e and S8a), nevertheless, 2H–MoTe_2_ can hardly photocatalytic CO_2_RR, in good accordance with above characterizations. In particular, the CO_2_-to-CH_4_ conversion rate of S_v_–In_2_S_3_@2H–MoTe_2_(5) reaches up to 70% (CH_4_ selectivity: 79.6%) with an optimum apparent quantum efficiency (AQE) value of 16.5% at 420 nm (Fig. S8b), which is comparable to most reported CO_2_-to-CH_4_ conversion rate at similar reaction conditions (Fig. S8c and Table S10). The remarkable CO_2_-to-CH_4_ conversion efficiency of S_v_–In_2_S_3_@2H–MoTe_2_(5) can mainly be due to the Mo–S bridging bonds and robust built-IEF. As depicted in Fig. S8d, the variation tendency of CO, H_2_, and CH_4_ production are consistent with characteristic absorption spectrum of S_v_–In_2_S_3_@2H–MoTe_2_(5), which strongly sustains that photocatalytic CO_2_RR is driven via the inter-band transition electrons of S_v_–In_2_S_3_@2H–MoTe_2_(5). Moreover, the control experiments in different conditions (Fig. 3c) confirm that the detected products are indeed derived from the reaction between CO_2_ and H_2_O, catalyzed by the samples, which is further affirmed via the result of ^13^CO_2_ labeling experiment in Fig. S9. Compared with pristine In_2_S_3_, S_v_–In_2_S_3_ with rich S-vacancies exhibits superior performance and excellent long-term stability. The photocatalytic performance of S_v_–In_2_S_3_@2H–MoTe_2_(5) was also tested in pure water. The production rate of CH_4_ decreased in pure water as compared with those (Fig. S10).

As displayed in Fig. 3f, the calculated data present higher values for AQE analysis of S_v_–In_2_S_3_@2H–MoTe_2_(5) at different wavelengths, compared with than that of 2H–MoTe_2_, In_2_S_3_, and S_v_–In_2_S_3_ (See Table S8 for details of the calculation results), further evaluating photocatalytic CO_2_RR activity. Moreover, S_v_–In_2_S_3_@2H–MoTe_2_(5) displays the highest AQE (65.29%) at 380 nm, being higher than most reported values (Table S9). Assisted by the robust built-IEF and Mo–S bonds of S_v_–In_2_S_3_@2H–MoTe_2_(5), “S”-scheme charge separation is notably facilitated, resulting in an IQE calculated via photocatalytic CO_2_RR of 94.01% (IQE_cr_) at 380 nm. This phenomenon proves the effective utilization of Mo–S bridging bonds and a breakthrough in IQE for S_v_–In_2_S_3_@2H–MoTe_2_(5) (Table S7). More specifically, cycling stability is also an important index to assessed the performance of photocatalyst in practical commercial use. Therefore, the stability of S_v_–In_2_S_3_@2H–MoTe_2_(5), S_v_–In_2_S_3_, 2H–MoTe_2_, and In_2_S_3_ was evaluated via cyclic stability tests in this work, respectively. As displayed in Fig. 3g, there is no remarkable decrease in evolution of CH_4_, CO, and H_2_, after 6 cycles of reaction using S_v_–In_2_S_3_@2H–MoTe_2_(5). According to the XRD patterns and TEM images (Fig. S11a–c) after 6 cycles test, the morphology and crystal structure of S_v_–In_2_S_3_@2H–MoTe_2_(5) have not changed significantly. Especially, XPS and EXAFS measurement was further used to study the change in chemical composition and atomic state before and after CO_2_RR (Figs. S11d, e and S12). Noteworthily, there is no peak shift in S_v_–In_2_S_3_@2H–MoTe_2_(5) after cyclic stability test. The above experimental results and analysis can prove outstanding morphology and structure of S_v_–In_2_S_3_@2H–MoTe_2_(5).

### Photoelectric Performance Analysis

**Fig. 4:**
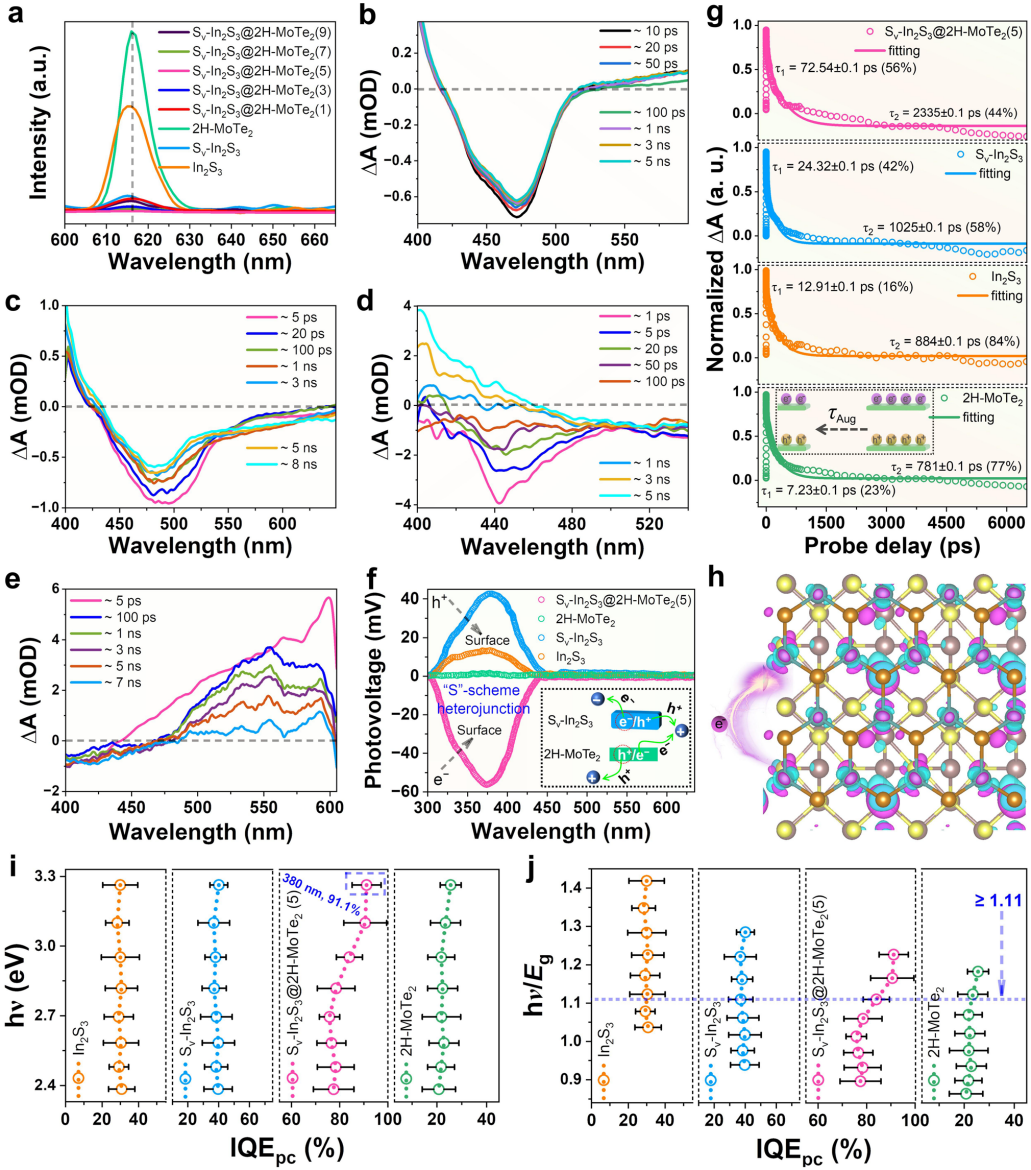
**a** PL spectra of samples (excitation wavelength = 300 nm). Fs-TA spectra of **b** S_v_–In_2_S_3_, **c** In_2_S_3_, **d** 2H–MoTe_2_, and **e** S_v_–In_2_S_3_@2H–MoTe_2_(5) measured at different delay times, respectively (320 nm excitation). **f** SPV spectra of samples. **g** Comparison of normalized exciton bleach signal decay between S_v_–In_2_S_3_ and 2H–MoTe_2_ (the curves were fitting results using Equation S1 and S2). **h** Differential charge density map of S_v_–In_2_S_3_@2H–MoTe_2_(5) (magenta area (positive value) and cyan area (negative value) represent accumulation and consumption of electrons, respectively). **i** IQE_pc_ as a function of illumination photon energy for samples.** j** IQE_pc_ as a function of multiples of bandgap (hυ/*E*_g_) of samples for samples (mean values with error bars showing s.d. for 3 measurements)

We evaluate the recombination of charge carriers through steady-state PL, ultrafast femtosecond transient absorption (fs-TA) spectroscopy, and TRPL spectra to study photocatalytic CO_2_RR activity. Clearly, 2H–MoTe_2_ exhibits strongest emission peak is corresponding to the rapid recombination of electrons-holes pairs (Fig. 4a), suggesting enhanced electronic conductivity of S_v_–In_2_S_3_@2H–MoTe_2_(5). Among them, S_v_–In_2_S_3_@2H–MoTe_2_(5) illustrates the weakest emission peak, consistent with their optimal photocatalytic CO_2_RR performance [36]. The fs-TA spectroscopic experiments were carried out on S_v_–In_2_S_3_@2H–MoTe_2_(5) exited at λ = 320 nm in CO_2_ atmosphere to further gain insight into electrons transfer dynamics during photocatalytic CO_2_RR process. For the purposes of comparison, we also recorded fs-TA spectra of S_v_–In_2_S_3_, In_2_S_3_, and 2H–MoTe_2_ under the same test condition. After excitation of S_v_–In_2_S_3_, a bleaching peak was observed at 474 nm, and the peak strength decreased with increasing delay time, which indicated that the recombination of a fraction of electrons and holes occurred in S_v_–In_2_S_3_ with prolonged delay time (Fig. 4b). Similar phenomenon is obtained for In_2_S_3_ (Fig. 4c), indicating that the relaxation process of S_v_–In_2_S_3_@2H–MoTe_2_(5) and S_v_–In_2_S_3_ under bandgap excitation is similar. For 2H–MoTe_2_, the strong and broad photoinduced bleaching peaks are observed at 442 nm, which is attributed to the generation of photoexcited holes in VB of 2H–MoTe_2_ [37, 38]. As delay time increased to 3 ns, these peaks are not observed in 2H–MoTe_2_ owning to the recombination of electrons and holes (Fig. 4d). Noteworthily, when S_v_–In_2_S_3_ was introduced into 2H–MoTe_2_, the peak strength of S_v_–In_2_S_3_ at 475 ~ 550 nm increased with increasing delay time (Fig. 4e, h), indicating that electrons transfer from 2H–MoTe_2_ to S_v_–In_2_S_3_ [37], which is completely consistent with the analysis results in Figs. S13–S15a. More specifically, the energy band structure of S_v_–In_2_S_3_ is in good agreement with that of 2H–MoTe_2_, and can attain the thermodynamic conditions for the spontaneous photocatalytic CO_2_RR process. The analysis of recovery kinetics discloses that the best decay fitting provides a bi-exponential function with two-time constants of *τ*_1_ = 72.54 ps and *τ*_2_ = 2335 ps for S_v_–In_2_S_3_@2H–MoTe_2_(5) (Fig. 4g). The *τ*_1_ and *τ*_2_ are attributed to the electron dynamics related to different electronic trap states with energies lie within the bandgap of S_v_–In_2_S_3_@2H–MoTe_2_(5). These two near-band edge trap states accumulate photogenerated electrons from bottom of CB in a bi-exponential relaxation (Fig. S15a) [39]. The longer carrier lifetime and stronger positive absorption of S_v_-In_2_S_3_@2H-MoTe_2_(5) are further evidenced via the Hall effect measurement. The Hall effect measurement at 300 K reveals that S_v_-In_2_S_3_@2H-MoTe_2_(5) possesses a carrier concentration of 4.51×10^13^ cm^−3^, an estimated carrier mobility of 2.38 cm^2^ V^−1^ s^−1^ (Table 1). The TRPL spectra in Fig. S15b and Table S6 display that S_v_–In_2_S_3_@2H–MoTe_2_(5) presents longer retention time (20.8 ns) of photoinduced carriers than that of S_v_–In_2_S_3_ (9.2 ns) due to the introduction of 2H–MoTe_2_ (179 ns), which further demonstrates the effective restraining effect to electrons-holes recombination. The carrier diffusion lengths (*L*_d_) are estimated to be in range of 0.26–0.36 μm for S_v_–In_2_S_3_@2H–MoTe_2_(5), and 0.025–0.05 μm for 2H–MoTe_2_, respectively (Table 1). Besides, the surface photovoltage (SPV) spectra were also conducted to validate its carrier transfer mechanism, as shown in Fig. 4f. It is noted that 2H–MoTe_2_ present no SPV signals in whole wavelength, illustrating the poor photocarrier separation efficiency. That’s why 2H–MoTe_2_ perform extremely poorly CO_2_RR activity. In comparison, a significant positive photovoltage response can be observed in SPV spectra of In_2_S_3_ and S_v_–In_2_S_3_, illustrating that the holes migrate to the surface of In_2_S_3_ and S_v_–In_2_S_3_, which is a typical trait of n-type semiconductors. Besides, in SPV spectra, unlike the positive photovoltage signal of 2H–MoTe_2_ and S_v_–In_2_S_3_, a negative and significantly enhanced photovoltage signal at 300–430 nm emerged in S_v_–In_2_S_3_@2H–MoTe_2_(5), demonstrating that the photogenerated electrons of S_v_–In_2_S_3_ and holes of 2H–MoTe_2_, transferred to illumination side and backlight side, respectively, and the remained electrons of 2H–MoTe_2_ recombined with holes of S_v_–In_2_S_3_ through a built-IEF, which further reveals the efficient interfacial charge transfer within the heterojunction via a “S”-scheme pathway.

**Table 1:** Carrier transport characteristics of samples

Samples	Bandgap (*E*_g_, eV)	Fs-TA lifetime (ps)	TRPL lifetime (ns)	Carrier concentration (n, cm^−3^)	Mobility [cm^2^ V^−1^ s^−1^]	Diffusion length [L_d_, μm]	IQE_cr_ (%)
S_v_–In_2_S_3_@2H–MoTe_2_(5)	2.66	τ_1_ = 12.3, τ_2_ = 2335	τ_1_ = 12.3, τ_2_ = 22.6	4.51 × 10^13^	2.38	0.26–0.36	94.01
S_v_–In_2_S_3_	2.54	τ_1_ = 24.32, τ_2_ = 1025	τ_1_ = 11, τ_2_ = 15	2.68 × 10^13^	1.64	0.2–0.24	12.88
In_2_S_3_	2.30	τ_1_ = 12.91, τ_2_ = 884	τ_1_ = 1.6, τ_2_ = 9.8	9.4 × 10^12^	0.99	0.06–0.15	11.76
2H–MoTe_2_	2.76	τ_1_ = 7.23, τ_2_ = 781	τ_1_ = 0.98, τ_2_ = 4.4	1.03 × 10^12^	0.27	0.025–0.05	10.51

^*^n.d. not determined

The incident photon-to-current efficiency (IPCE) and corresponding IQE_pc_ were measured under different wavelengths of monochromatic light irradiation to investigate the photoelectric conversion efficiency of S_v_–In_2_S_3_@2H–MoTe_2_(5) [40]. The IPCE profiles of S_v_–In_2_S_3_@2H–MoTe_2_(5) at different wavelengths are consistent with the above optical absorption results (Fig. S15c), verifying excellent carrier transfer and separation. The IQE_pc_ was assessed by normalizing the IPCE values to the measured absorption curve of S_v_–In_2_S_3_@2H–MoTe_2_(5), S_v_-IS SSNBs, In_2_S_3_, and 2H–MoTe_2_. The IQE_pc_ curves of S_v_–In_2_S_3_, In_2_S_3_, and 2H–MoTe_2_ remain flat in total wavelength range (380–520 nm), while the IQE_pc_ curves of S_v_–In_2_S_3_@2H–MoTe_2_(5) show an upward trend when S_v_–In_2_S_3_ and 2H–MoTe_2_ were combined to form Mo–S bridging bonds (Fig. 4i, j). Intriguingly, the S_v_–In_2_S_3_@2H–MoTe_2_(5) discloses the maximum IQE_pc_ value of 91.1% at 320 nm, indicating enhanced exciton extraction force driven by a strong built-IEF at the interface between S_v_–In_2_S_3_ and 2H–MoTe_2_. To further validate our perception, the IQE_pc_ curves vs. *E*_g_ of S_v_–In_2_S_3_@2H–MoTe_2_(5), S_v_–In_2_S_3_, In_2_S_3_, and 2H–MoTe_2_ are plotted to elucidate the photon absorption and conversion in S_v_–In_2_S_3_@2H–MoTe_2_(5). The IQE_pc_ of S_v_–In_2_S_3_@2H–MoTe_2_(5) gradually increases when the incident photon energy exceeds 1.11 times the *E*_g_ of 2H–MoTe_2_, significantly promoting charge transport and separation within S_v_–In_2_S_3_@2H–MoTe_2_(5), which is mainly due to possibility of multiple exciton production in 2H–MoTe_2_ [41].

### Relationship Between Mo–S Bridging and CO_2_RR Activity

**Fig. 5:**
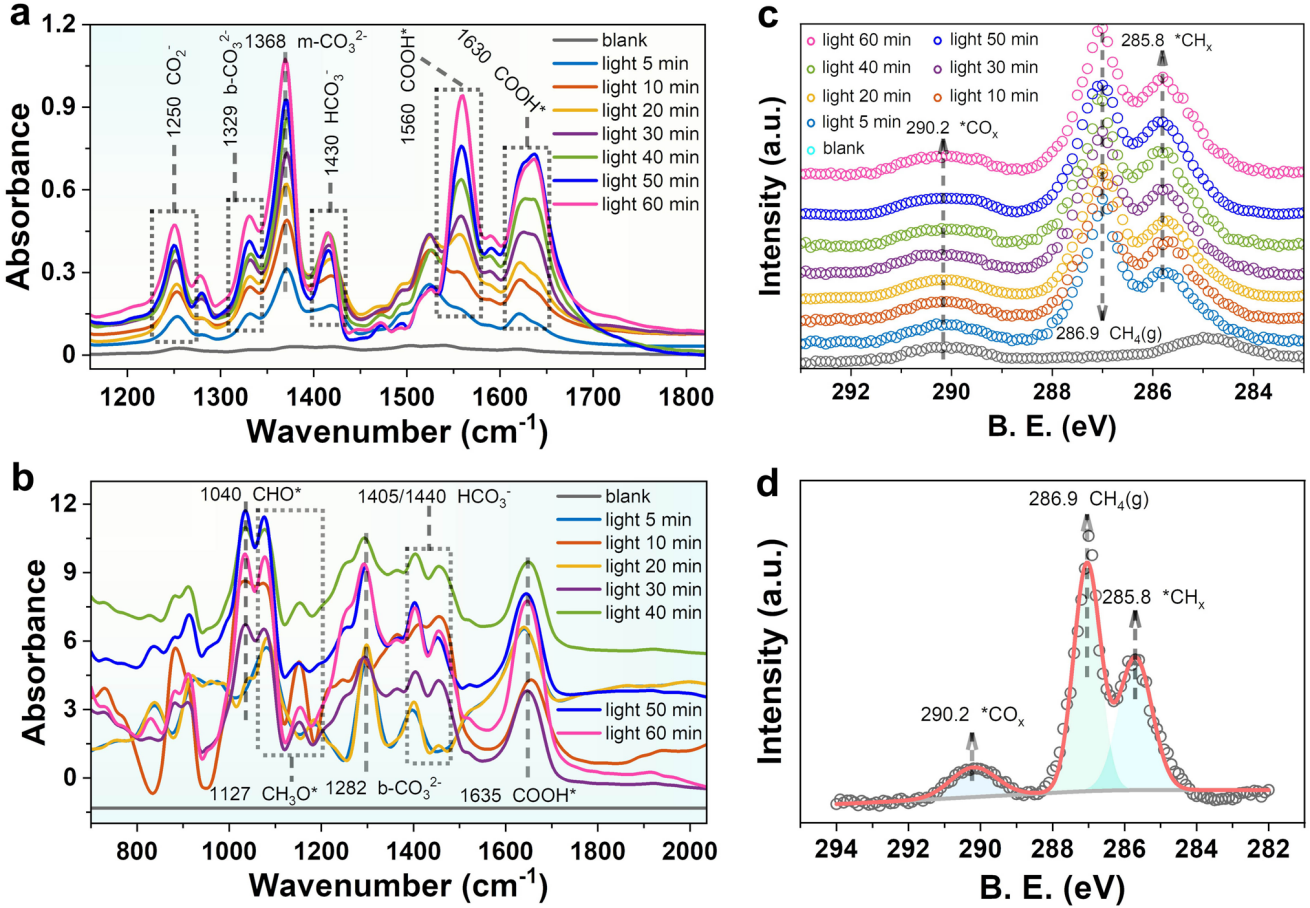
In situ DRIFTS spectra for photocatalytic CO_2_RR over **a** S_v_–In_2_S_3_@2H–MoTe_2_(5) and **b** S_v_–In_2_S_3_. **c** In situ high-resolution C 1*s* XPS spectra of S_v_–In_2_S_3_@2H–MoTe_2_(5) with different light illumination time. **d** C 1*s* near ambient pressure XPS (NAP-XPS) collected for CH_4_ conversion over S_v_–In_2_S_3_@2H–MoTe_2_(5) under light illumination at 5 min

In situ diffuse reflectance–Infrared Fourier transform spectroscopy (DRIFTS) and in situ high-resolution XPS spectroscopy were performed to correlate surface characteristics to the efficiency of photocatalytic CO_2_RR progress. The analysis results are shown in Fig. 5. From 0 to 60 min, new absorption peaks perceptibly appear with increasing light time and their intensity gradually increase. The observation of new infrared peak at 1127 cm^−1^ gradually increases, which can be ascribed to the CH_3_O* intermediates (the asterisk denotes the catalytically active sites), while the peak at about 1040 cm^−1^ can be assigned to the characteristic bands of CHO*. The peaks at 1560 and 1630 cm^−1^ are attributed to the COOH*, which is generally regarded as the key intermediates of CO_2_ photoreduction to CH_4_ or CO, as well CH_3_OH [42, 43]. The peaks at 1430 cm^−1^ are corresponded to symmetric stretching of HCO_3_*, respectively. The formation of monodentate carbonate (m-CO_3_^2−^) and bidentate carbonate (b-CO_3_^2−^) are evidenced from infrared peaks of around 1368 and 1329 cm^−1^, respectively (Fig. 5a) [44, 45]. Similar phenomenon is obtained for S_v_–In_2_S_3_ (Fig. 5b). The in situ XPS was used to study changes of hydrocarbons on the surface of S_v_–In_2_S_3_@2H–MoTe_2_(5) during photocatalytic CO_2_RR process. In dark state, no peak of gas-phase CH_4_ (286.9 eV) is observed (Fig. 5c, d), illustrating that photoreduction of CO_2_–CH_4_ is light-driven. In contrast, with the gradual increase of light, a peak of surface-CH_x_ species appears at about 285.8 eV, further supporting the dissociation of generated CH_4_ at the surface of catalyst to form H_2_, which is why we detected the presence of H_2_ product in mixing products.

**Fig. 6:**
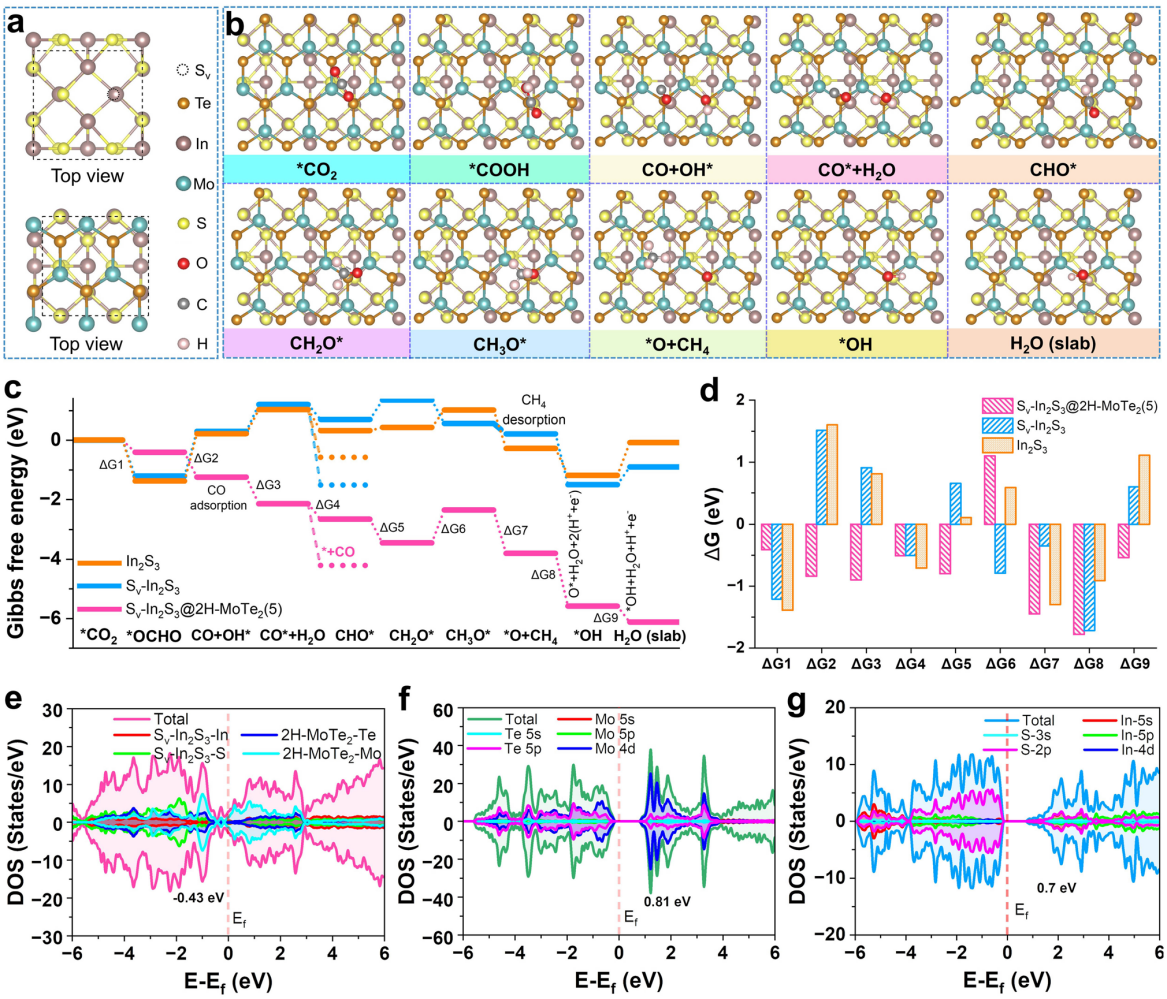
**a** Atomic models of S_v_–In_2_S_3_@2H–MoTe_2_(5) in theoretical calculations. **b** Schematic illustration of adsorption atomic structures during CO_2_RR process on over S_v_–In_2_S_3_@2H–MoTe_2_(5) interfaces.** c** Schematic Gibbs energy profiles and** d** energy changes for CO_2_RR pathway at 1.23 V on different active sites for S_v_–In_2_S_3_@2H–MoTe_2_(5), S_v_–In_2_S_3_, and In_2_S_3_, respectively. The calculated DOS of** e** S_v_–In_2_S_3_@2H–MoTe_2_(5),** f** 2H–MoTe_2_, and **g** S_v_–In_2_S_3_

In terms of the above analyses, the DFT simulations were performed to gain in-depth insights into Mo–S bridging bonds mechanism toward CO and CH_4_ products on S_v_–In_2_S_3_@2H–MoTe_2_(5). In this research, in order to more accurately explain thermodynamic process of photoreduction of CO_2_–CO, H_2_, and CH_4_, we introduced S-vacancies into structure of In_2_S_3_ under visible-light irradiation and determined the most optimal position for S-vacancies to participate in photocatalytic reaction (Fig. S22). Initially, CO_2_ will be gradually adsorbed on the surface of S_v_–In_2_S_3_@2H–MoTe_2_(5), and H_2_O in solution will be concomitantly dissociated and produce hydroxide ions (OH^¯^) and H^+^. It is worth noting that path 1 is an endothermic during the reaction (Δ*G* > 0), so it is not considered here (Fig. S23). The Gibbs free energy analysis curves for photocatalytic CO_2_-to-CH_4_ process (path 2) with the lowest energy pathway on the surface of S_v_–In_2_S_3_@2H–MoTe_2_(5) was calculated in detail, as shown in Fig. 6. During reaction process of photocatalytic CO_2_RR to form CO, H_2_, and CH_4_, seven intermediate products can be produced, which are *OCHO, OH*, CHO*, CH_2_O*, CO*, O*, and CH_3_O*, respectively. The other *CO on the surface diffuse toward S-vacancies and couple with those reaction intermediates to produce CH_4_ (Fig. 6a, b). The CH_4_ free energy diagrams are summarized in Fig. 6c, while the corresponding minimum energy reaction pathways are presented in Fig. 6d. The diagram of free energy calculations illustrates that the reaction process of *OCHO–CO(g) and *OH is a potentially decisive step (Δ*G* = ** − **0.84 eV). Initially, the CO_2_ energetically favor Mo–S bridging bonds sites from CO_2_–CO. When one H atom approaches the adsorbed CO_2_, it can form *OCHO [46]. Noteworthily, the S-vacancies can promote activation of CO_2_, reduce energy barrier for the formation of *OCHO, and promote charge transfer to *OCHO, thereby promoting CO_2_RR to form CO [47]. Furtherly, the formation of *OCHO is the step with the highest energy barrier in the formation of final CH_4_, and thus, the *OCHO will transition to OH*. The ΔG of CO* desorption is around − 0.51 eV lower than that of CHO* (Fig. 6c and Fig. S18a), resulting in a mixture of final products with CO, H_2_, and CH_4_ at Mo–S bridging bonds sites during CO_2_RR process. It should be emphasized that CO desorption on S_v_–In_2_S_3_@2H–MoTe_2_(5) is an exothermic process. In contrast, the hydrogenation of CO*–CHO* is spontaneously exothermic, namely Δ*G* < 0, resulting in a better selectivity for photoreduction of CO_2_–CO, which perfectly accords with above results analysis of the CO_2_-TPD (Fig. 3a).

We also explored reaction energy barriers of S_v_–In_2_S_3_@2H–MoTe_2_(5) for H_2_O splitting under alkaline conditions. As shown in Fig. S18a–c, we constructed the reaction path of alkaline HER, including previous water dissociation to the formation of H* (Volmer step) and H_2_ generation (Tafel step or Heyrovsky step). The 2H–MoTe_2_ shows the highest H**–**OH adsorption energy (Δ*G*_ads_ = 1.21 eV) and H* adsorption energy barrier (Δ*G*_H*_ = 0.55 eV), suggesting that the strong H* adsorption energy of In_2_S_3_ will hinder the evolution of H_2_, resulting in slow HER kinetics, which is consistent with the experimental results (Fig. 3e). The d-band center position of catalysts is an important factor that determines the adsorption energy of intermediates. Significantly, the combined analysis of free energy and density of state (DOS) calculations and electrostatic potentials simulation present apparent evidence for step-by-step reactions of S_v_–In_2_S_3_, In_2_S_3_, and S_v_–In_2_S_3_@2H–MoTe_2_(5) promoted via modulation of active sites and electronic structures [48]. An upshifted d-band center toward Fermi level reveals enhanced adsorption of intermediates. This is duo to the higher energy level of the d-band center allows for stronger interaction between photocatalyst and intermediates, leading to more efficient photocatalytic CO_2_RR [49]. The S_v_–In_2_S_3_@2H–MoTe_2_(5) has a significantly upshifted d-band center (**− **0.43 eV) compared to S_v_–In_2_S_3_ (0.70 eV) and 2H–MoTe_2_ (0.81 eV) (Fig. 6e − g), illustrating that S_v_–In_2_S_3_@2H–MoTe_2_(5) should possess a stronger binding strength for CO_2_RR intermediates. Herein, we demonstrate that the introduction of Mo–S bridging bonds to construct heterogeneous structures tailors the d-band center, which in turn affects the adsorption capacities of different intermediates (Scheme 1) and ultimately optimizes CO_2_RR activity. The cyan and magenta regions indicate electron depletion and accumulation (Figs. 4 h and S14d), respectively. This result can be derived from influence of S-vacancies on the electronic structure of enhanced damage prevention in S_v_–In_2_S_3_@2H–MoTe_2_(5) interfaces.

## Conclusion

In summary, inspired by the construction of a strong IEF that can elevate d-band center to Fermi level, we elaborately designed a double-shelled nanoboxes structure, an ultrathin 2H–MoTe_2_ coated S_v_–In_2_S_3_ to form Mo–S bridging bonds sites for CO_2_RR. The in situ characterization and DFT calculations affirmed that a strong interfacial electric field of S_v_–In_2_S_3_@2H–MoTe_2_(5) can reduce adsorption energy barriers of *OCHO and *CHO, and significantly enhance reaction rate of the rate-determining step on the surface of Mo–S bridging bonds. The S-vacancies can promote activation of CO_2_, reduce energy barrier for the formation of *OCHO, and promote charge transfer to *OCHO, thereby promoting CO_2_RR to form CO. Furthermore, the charge difference leads to the formation of polarization sites of Mo at the interface, which inhibits the electrostatic repulsion of adjacent intermediates and promotes formation of CO and CH_4_.This study reveals that the interfacial electric field in S_v_–In_2_S_3_@2H–MoTe_2_(5) can obviously facilitate CO_2_RR via tuning the d-band center of Mo and adsorption of intermediates, which provides a guideline for future rational fabrication and construction of catalysts for CO_2_RR and other related reactions.

## Supplementary Information

Below is the link to the electronic supplementary material.

Supplementary file 1 (2174 PDF)
