# Supplementary material for: Understanding Bridging Sites and Accelerating Quantum Efficiency for Photocatalytic CO2 Reduction
Source: Nanomicro Lett. 2023 Nov 6;16:5. doi: 10.1007/s40820-023-01221-3 (PMC10628097; doi:10.1007/s40820-023-01221-3)
Supplement: Supplementary file 1 — (2174 PDF) [file 40820_2023_1221_MOESM1_ESM.docx]

**Supporting Information**

**Understanding** **Bridging Sites and** **Accelerating** **Quantum Efficiency for Photocatalytic CO_2_ Reduction**

Kangwang Wang^1^, Zhuofeng Hu^2^, Peifeng Yu^1^, Alina M. Balu^3^, Kuan Li^1^, Longfu Li^1^, Lingyong Zeng^1^, Chao Zhang^1^, Rafael Luque^4,5^, Kai Yan^2*^, Huixia Luo^1,^ *

^1^ School of Materials Science and Engineering, State Key Laboratory of Optoelectronic Materials and Technologies, Guangdong Provincial Key Laboratory of Magnetoelectric Physics and Devices, Key Lab of Polymer Composite & Functional Materials, Sun Yat-Sen University, No. 135, Xingang Xi Road, Guangzhou, 510275, P. R. China

^2^ School of Environmental Science and Engineering, Sun Yat-Sen University, No. 135, Xingang Xi Road, Guangzhou 510275, P. R. China

^3^ Departamento de Química Orgánica, Universidad de Córdoba, Campus Universitario de Rabanales, Edificio Marie Curie (C3), Córdoba E-14014, Spain

^4^ Center for Refining and Advanced Chemicals, King Fahd University of Petroleum and Minerals, Dhahran 31261, Saudi Arabia

^5^ Universidad ECOTEC, Km 13.5 Samborondón, Samborondón EC092302, Ecuador

*Corresponding author. E-mail: [luohx7@mail.sysu.edu.cn](mailto:luohx7@mail.sysu.edu.cn) (Huixia Luo); [yank9@mail.sysu.edu.cn](mailto:yank9@mail.sysu.edu.cn) (Kai Yan)

**Section I: Experiment section**

**Synthesis**

***Preparation of Cu_2_O nanocubes (NCs):***

Cu_2_O NCs were prepared by adapted from known literature procedure [^[[1]](#endnote-2)^,^[[2]](#endnote-3)^]. Specifically, CuSO_4_∙5H_2_O (1.5 mmol) and sodium citrate (0.5 mmol) were dissolved into 80 mL of deionized water (DW). Then, 20 mL NaOH (1.25 M) was added to the above solution under stirring. Afterward, 50 mL ascorbic acid (0.03 M) was added to the suspension under stirring for 5 min. The obtained mixed solution was stood for 1 h at room temperature. The precipitate was collected by centrifugation and washed with DW and ethanol several times.

***Preparation of Cu_2_O@In(OH)_3_ core-shell nanocubes (CSNCs):***

A quarter of the as-prepared Cu_2_O NCs and 11 mg of InCl_3_ were first dispersed into 40 mL of ethanol/H_2_O solvent (volume ratio = 1:1) in the presence of 1.33 g of PVP by sonication for 10 min. Then, a Na_2_S_2_O_3_ aqueous solution (1.0 M, 16 mL) was added to the above mixture under stirring. After 1.5 (4.0, 6.0, 8.0, and 10.0) min of reaction, the precipitate was collected by centrifugation and washed with DW and ethanol several times, respectively.

***Preparation of In(OH)_3_*** ***single-shelled nanoboxes (SSNBs):***

The as-prepared Cu_2_O@In(OH)_3_ CSNCs were dispersed into 20 mL of ethanol/H_2_O solvent (volume ratio = 1:1). Then, a Na_2_S_2_O_3_ aqueous solution (1.0 M, 8 mL) was added to the above mixture under stirring. After 30 min reaction, the precipitate was collected by centrifugation and washed with DW and ethanol several times.

***X-ray Absorbtion Spectra (XAS)****.* The X-ray absorption fine structure (XAFS) spectroscopy of Mo K-edge was collected at the X-ray absorption fine structure for catalysis (XAFCA) beamline of the Singapore Synchrotron Light Source, Singapore. Extended X-ray absorption fine structure (EXAFS) fitting was applied through Athena and Artemis software [^[[3]](#endnote-4)^]. Wavelet transformation (WT) was also employed using the software package developed by Funke and Chukalina using Morlet wavelet with *κ* = 10, *σ* = 1 [^[[4]](#endnote-5)^,^[[5]](#endnote-6)^]. *S*_0_^2^ was obtained from Mo foil and fixed as 0.92. Δ*E*_0_ was returned a value of 2.2206 ± 0.4129 eV (3.0 ≤ *k* ≤ 12.0 Å^−1^, 1.3 ≤ *R* ≤ 2.8 Å). The number of variable parameters was 3 out of a total of 8.3906 independent data points, and the *R* factor for this fit was 1.06%. The Debye-Waller factors were obtained based on the guessing parameters and constrained as 0.004 and 0.002 for Mo**−**S and Mo**−**Te, respectively. The delta *R*_s_ was based on guessing parameters and fixed as **−**0.003 and **−**0.02 for Mo**−**S and Mo**−**Te, respectively.

***UPS spectra.*** The work function (*Φ*) of samples can be obtained by subtracting the cutoff binding energy (*E*_Cutoff_) from the radiation photon energy (He I α at 21.2 eV), while the valence band (*VB*) edge and *Φ* have the following relationship:

| $\Phi=h\nu-E_{\text{Cutoff}}$ | ES1 |
| --- | --- |
| $VB=\Phi-E_{\text{VBM}}=h\nu-E_{\text{Cutoff}}-E_{\text{VBM}}$ | ES2 |

The bandgaps energy of In_2_S_3_, S_v_-In_2_S_3_, 2H-MoTe_2_, and S_v_-In_2_S_3_@2H-MoTe_2_(5) were calculated by the following equation (Kubelka-Munk (KM) equation):

| $\alpha h\upsilon=A\left(h\upsilon-E_{g}\right)^{n/2}$ | ES3 |
| --- | --- |

in which *α*, *h*, *υ*, *A*, and *E*_g_ represent the absorption coefficient, Planck constant, light frequency, proportionality, and bandgaps energy, respectively. Combining the *E*_g_ values from the UV-Vis absorption spectrum, the conduction band (*CB*) edge of the nanofiber films was related to *VB* as follows:

| $VB=CB+E_{\text{g}}$ | ES4 |
| --- | --- |

***TRPL analysis.*** In terms of TRPL, the decay curves can be well fitted by the following biexponential equations [^[[6]](#endnote-7)^,^[[7]](#endnote-8)^]:

| $I_{\left(t\right)}=I_{0}+A_{1}exp(-t\text{/}\tau_{1})+A_{2}exp(-t\text{/}\tau_{2})$ | ES5 |
| --- | --- |
| $\tau_{a}=(A_{1}\tau_{1}^{2}+A_{2}\tau_{2}^{2})\text{/}(A_{1}\tau_{1}+A_{2}\tau_{2})$ | ES6 |

where *I*_0_ represents the baseline correction value, *A*_1_ and *A*_2_ are the pre-exponential factors. *τ*_1_, *τ*_2_, and *τ*_a_ represent the lifetime (ns) in different processes, and average lifetime.

***Bleach decay kinetics fitting.*** The bleach decay kinetics in Fig. 4g (320 nm excitation) were fitted with the following equations:

| $\Delta A\left(t\right)=\sum_{i=1}^{\text{i}} A_{i}\exp(-\frac{t}{\tau_{rec_{i}}})\otimes G(\Delta t)$ | ES7 |
| --- | --- |
| $\Delta A=e^{-(\frac{t}{\tau_{x}})^{\beta}}$ | ES8 |

***Carrier concentration and carrier diffusion lengths (L_d_):***

| $\text{n}=\text{ }\frac{1}{\left\vert R_{\text{h}}\right\vert}=\text{ }\frac{1}{\left\vert\frac{Ud}{IB}\right\vert}$ | ES9 |
| --- | --- |
| $L_{d}=\text{ }\left(\frac{k_{\text{B}}T}{e}\times\mu\times\tau\right)^{\frac{1}{2}}\text{ }$ | ES10 |

where *U* is the Hall voltage, expressed in mv; *d* is the thickness of the Hall element, expressed in μm; *I* is the working current, expressed in mA; and *B* is the magnetic field strength, expressed in T. *k*_B_ is the Boltzmann’s constant, *T* is the absolute temperature, *μ* is the carrier mobility, and *τ* is the carrier lifetime (Table 1).

***CO_2_ temperature programmed desorption (CO_2_-TPD) measurements****.* Typically, S_v_-In_2_S_3_, 2H-MoTe_2_, and S_v_-In_2_S_3_@2H-MoTe_2_(5) were maintained at 150 ℃ for 1 h in a vacuum environment. Subsequently, the samples were cooled down to room temperature, followed by the exposure of CO_2_ under 1.0 mbar. After pumping excess CO_2_ gas, the samples were heated up to 350 ℃ at the rate of 2 ℃·min^−1^ to measure the desorption spectra during the detection process [^[[8]](#endnote-9)^].

***Incident photon-to-current efficiency (IPCE) and corresponding*** ***internal quantum efficiency (IQE_pc_).*** The remarkable photoelectric conversion capacity of In_2_S_3_, S_v_-In_2_S_3_, 2H-MoTe_2_, and S_v_-In_2_S_3_@2H-MoTe_2_(5) was also revealed in IPCE profile (Fig. S15c). The detailed IPCE and corresponding IQE_pc_ calculation process via photocurrent measurement is based on equations:

| $\text{IPCE}=(\frac{1240\cdot J_{\text{p}}}{\lambda\cdot I_{\text{light}}})\times100\%$ | ES11 |
| --- | --- |
| $\text{IQE}_{\text{pc}}=\frac{\text{IPCE}}{A}=(\frac{1240\cdot J_{\text{p}}}{\lambda\cdot I_{\text{light}}\cdot A})\times100\%$ | ES12 |

where *J*_p_ is the photocurrent density, λ represents the wavelength of the monochromatic light, *I*_light_ refer to the incident light power density. The A is the absorbance of the photocatalysts layer on the surface of sample.

***Apparent quantum efficiency (AQE) and corresponding IQE (IQE_cr_).*** The AQE was determined under above photocatalytic reaction conditions except that five LEDs (420 nm, 3 W) were used as irradiation source. The wavelength-dependent apparent quantum efficiency was measured under the same photocatalytic reaction condition, except for the monochromatic light wavelengths (380, 400, 420, 440, 460, 480, 500, and 520 nm). All the experiments were repeated at least 3 times in parallel to obtain an average value. At the same time, AQE and IQE_cr_ was roughly calculated from the equation [^[[9]](#endnote-10)^]:

| $\begin{matrix} \text{AQE = }\frac{\text{the number of reacted electrons}}{\text{the number of incident photons}}\text{ }\times\text{ 100\% } \\ \\ \text{ = }\frac{\text{8 }\times\text{ the number of evolved CH}_{4}\text{molecules}}{\text{N}}\text{ }\times\text{ 100\% } \\ \\ \text{ = }\frac{\text{8 }\times\text{ the number of evolved CH}_{4}\text{molecules}}{\frac{E\lambda}{hc}}\text{ }\times\text{ 100\%} \end{matrix}$ | ES13 |
| --- | --- |
| $\text{IQE}_{\text{cr}}\text{= }\frac{\text{AQE}}{A}\text{ = }\frac{\text{8 }\times\text{ the number of evolved CH}_{4}\text{molecules}}{\frac{AE\lambda}{hc}}\text{ }\times\text{ 100\% }$ | ES14 |

where *A* is the absorption of sample solution.

***Evaluation of Catalytic Performance.*** CO_2_ conversion was calculated according to the following equation:

|  | ES15 |
| --- | --- |

Product selectivity was calculated on a molar carbon basis. Product selectivity was calculated according to the following equation:

| $S_{i}\text{ }=\text{ }\frac{C_{i}\times n_{i}}{\sum(C_{i}\times n_{i})}\text{ }\times\text{ }100\%$ | ES16 |
| --- | --- |

where *C_i_* indicates the molar fraction of product *i* and *n_i_* is the carbon number of products *i*.

***d-band center.*** To get the individual orbital components, we employed the code-named “splitdos.dos” to process the output file of the density of states (DOS). The average *d*-band (*d* band center) shifts are calculated for the surface metal atoms for both the total *d* partial DOS and the orbital-resolved *d* partial DOS. The *d* band center (𝜀_d_) is calculated as:

| $\varepsilon_{\text{d}}\text{=}\frac{\int n_{d}(\varepsilon)\varepsilon d\varepsilon}{\int n_{d}(\varepsilon)d\varepsilon}$ | ES17 |
| --- | --- |

where 𝜀 is the electronic energy of states, and 𝑛_d_(𝜀) is the electronic density of states [^[[10]](#endnote-11)^].

***Work function.*** The following equation can calculate the work function (*Φ*) of a material: *Φ* = *E*_vac_−*E*_F_, *E*_vac_ and *E*_F_ are the vacuum energy and fermi level energy, respectively. In addition, the band structures and DOS of S_v_-In_2_S_3_, 2H-MoTe_2_, and S_v_-In_2_S_3_@2H-MoTe_2_(5) interface were calculated.

**Section Ⅱ: Figures and tables**


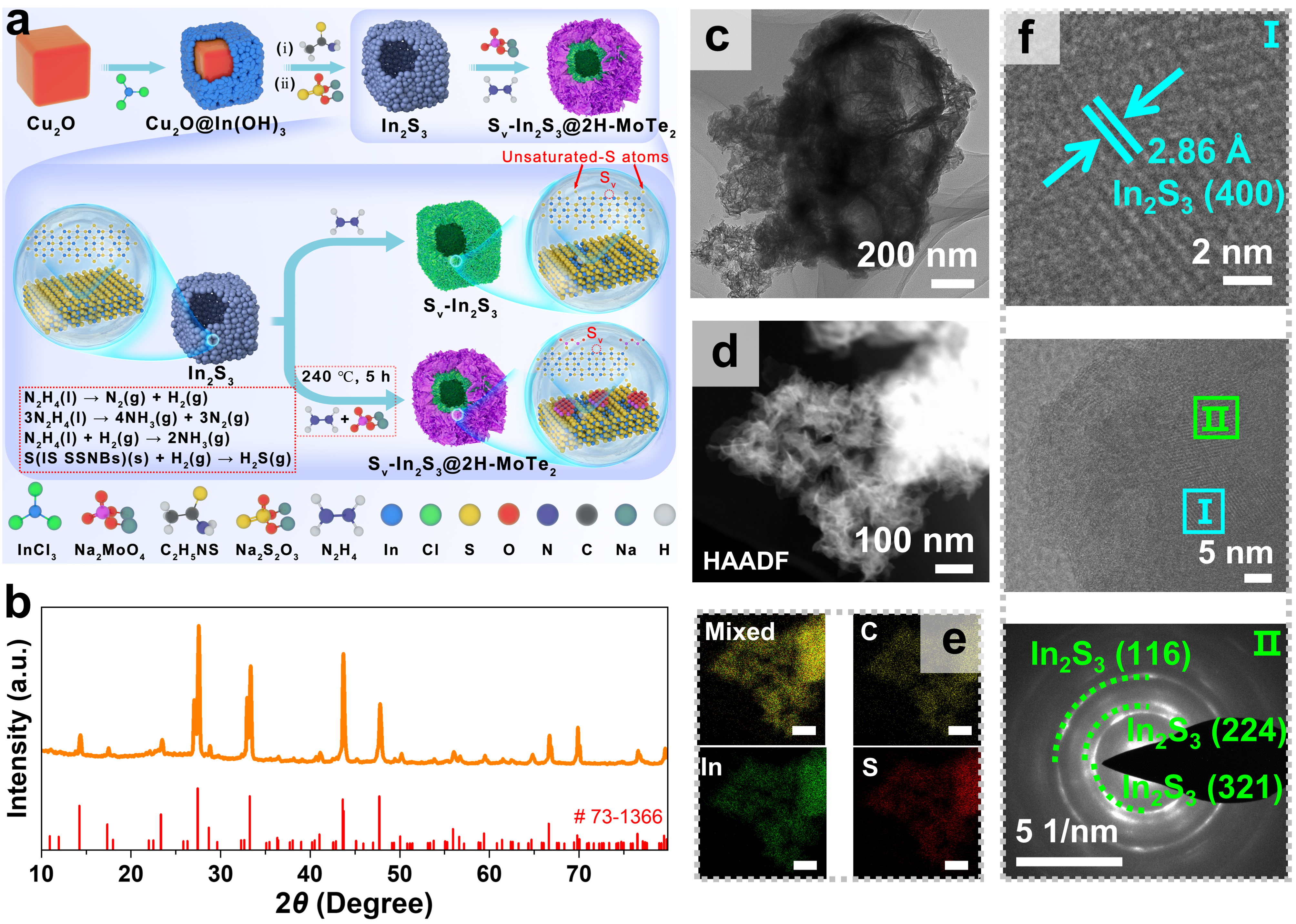


**Fig. S1** **a** Schematic diagram of preparation process of S_v_-In_2_S_3_@2H-MoTe_2_(5). **b** XRD pattern, **c** TEM images, **d** HAADF-STEM and **e** EDX mapping images (Scale bar = 50 nm), **f** HRTEM images and SAED pattern of In_2_S_3_.


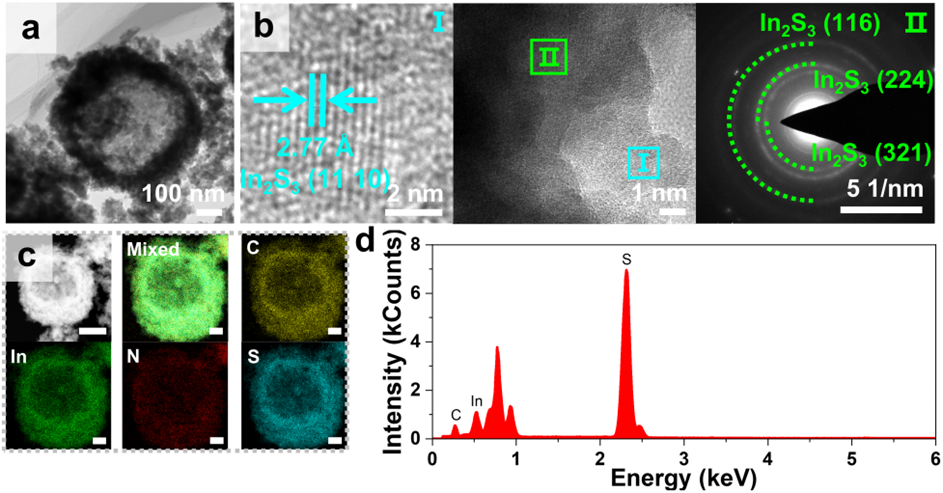


**Fig. S2** **a** TEM, **b** HRTEM, **c** HAADF-STEM and EDX mapping images (Scale bar = 50 nm), and **d** EDX spectrum of S_v_-In_2_S_3_.


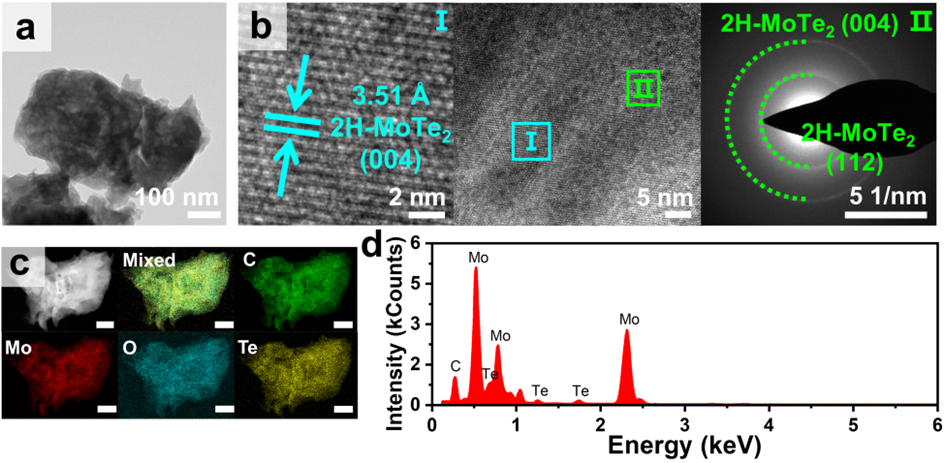


**Fig. S3** **a** TEM, **b** HRTEM and SEAD pattern, **c** HAADF-STEM and EDX mapping images (Scale bar = 100 nm), and **d** EDX spectrum of 2H-MoTe_2_.

**Table S1** Elemental analysis of S_v_-In_2_S_3_@2H-MoTe_2_(5) by EDX spectrum.

| Element | Atomic (%) | Molecular formula |
| --- | --- | --- |
| In | 22.15 | In_1.00_S_1.48_ |
| S | 32.85 |  |
| Mo | 14.74 | Mo_1.00_Te_2.05_ |
| Te | 30.16 |  |


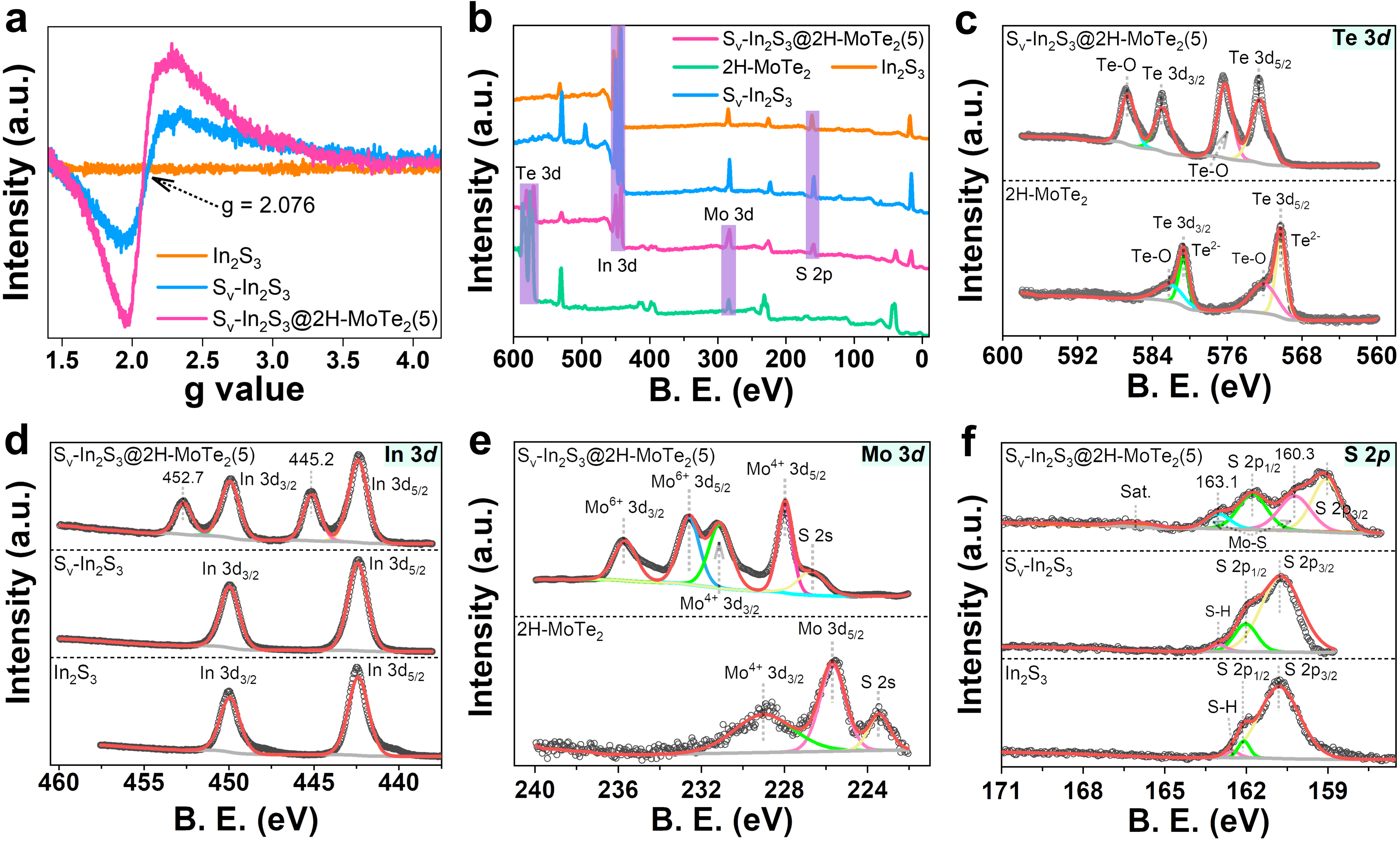


**Fig. S4 a** EPR spectra of In_2_S_3_, S_v_-In_2_S_3_, and S_v_-In_2_S_3_@2H-MoTe_2_(5), respectively. **b** XPS full-scan survey spectra of 2H-MoTe_2_, In_2_S_3_, S_v_-In_2_S_3_, and S_v_-In_2_S_3_@2H-MoTe_2_(5), respectively. The high-resolution XPS spectra of **c** Te 3d, **d** In 3d, **e** Mo 3d, and **f** S 2p in 2H-MoTe_2_, In_2_S_3_, S_v_-In_2_S_3_, and S_v_-In_2_S_3_@2H-MoTe_2_(5), respectively.

**Table S2** Elemental analysis of as-prepared photocatalysts via XPS spectra.

| Samples | S_v_-In_2_S_3_@2H-MoTe_2_(5) | | | | S_v_-In_2_S_3_ | | In_2_S_3_ | | 2H-MoTe_2_ | |
| --- | --- | --- | --- | --- | --- | --- | --- | --- | --- | --- |
| Method | In/% | S/% | Mo/% | Te/% | In/% | S/% | In/% | S/% | Mo/% | Te/% |
| XPS | 12.65 | 19.1 | 3.07 | 5.45 | 20.64 | 30.61 | 21.7 | 28.29 | 15.43 | 36.13 |


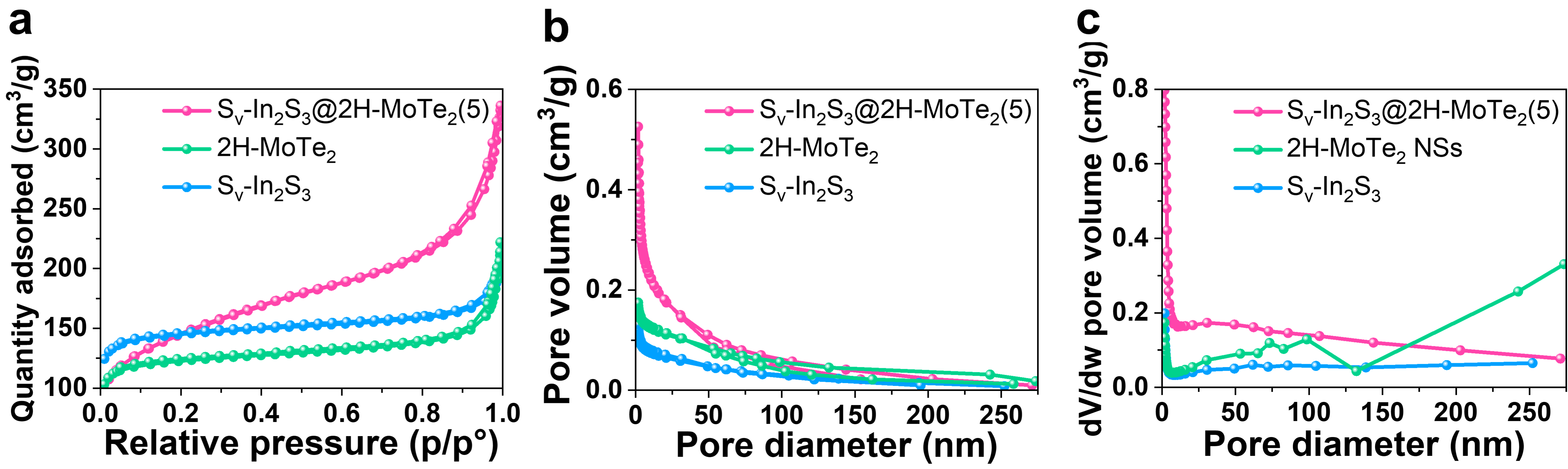


**Fig. S5** **a** Nitrogen adsorption-desorption isotherms, **b** cumulative intrusion curves, and **c** BJH pore-size distribution of S_v_-In_2_S_3_, 2H-MoTe_2_, and S_v_-In_2_S_3_@2H-MoTe_2_(5), respectively.

**Table S3** Surface area, pore diameter, and pore volume for 2H-MoTe_2_, S_v_-In_2_S_3_, and S_v_-In_2_S_3_@2H-MoTe_2_(5), respectively.

| Samples | BET surface area (m^2^·g^−1^) | Pore volume (cm^3^·g^−1^) | Pore diameters (nm) |
| --- | --- | --- | --- |
| 2H-MoTe_2_ | 395.46 | 0.175 | 8.20 |
| S_v_-In_2_S_3_ | 465.95 | 0.127 | 5.93 |
| S_v_-In_2_S_3_@2H-MoTe_2_(5) | 495.44 | 0.526 | 4.51 |


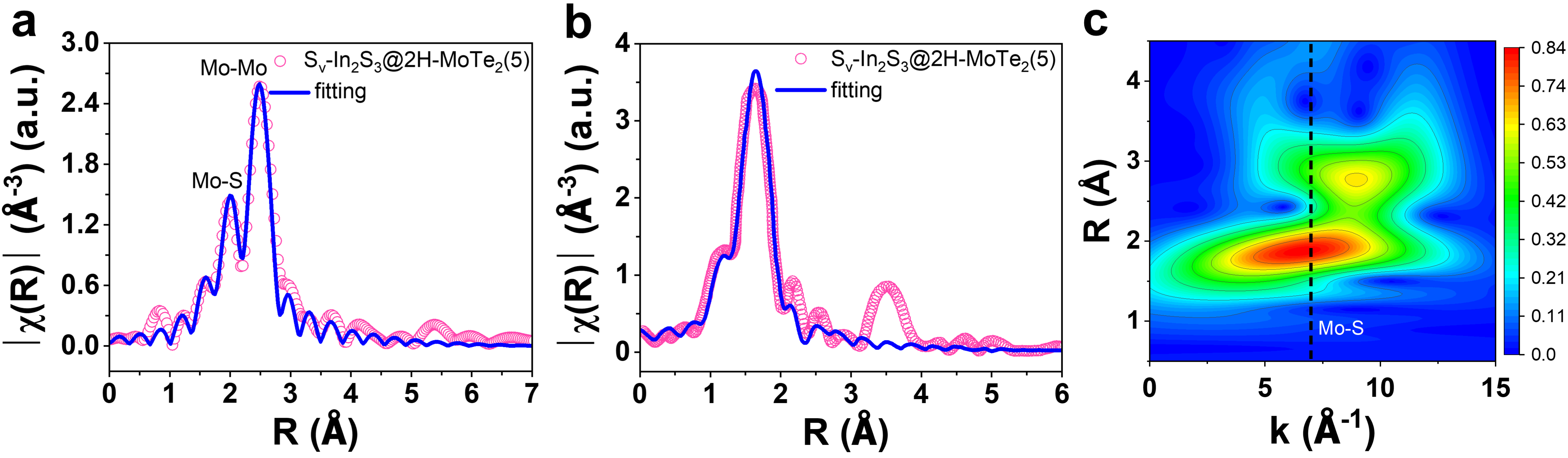


**Fig. S6** **a** Mo and **b** K-edge EXAFS (points) and fit (line) for S_v_-In_2_S_3_@2H-MoTe_2_(5), shown in k^2^ weighted R-space. **c** WT for the k^3^ weighted EXAFS contour plots of MoS_2_.

**Table S4** Fitting parameters for Mo K-edge EXAFS for S_v_-In_2_S_3_@2H-MoTe_2_(5).

| Paths | CN | R (Å) | σ^2^ (10^−3^ Å^2^) | Δ*E*_0_ |
| --- | --- | --- | --- | --- |
| Mo**−**S | 1.4±0.3 | 2.41 | 0.004 | 2.2206 ± 0.4129 |
| Mo**−**Te | 4.1±0.2 | 2.72 | 0.002 |  |


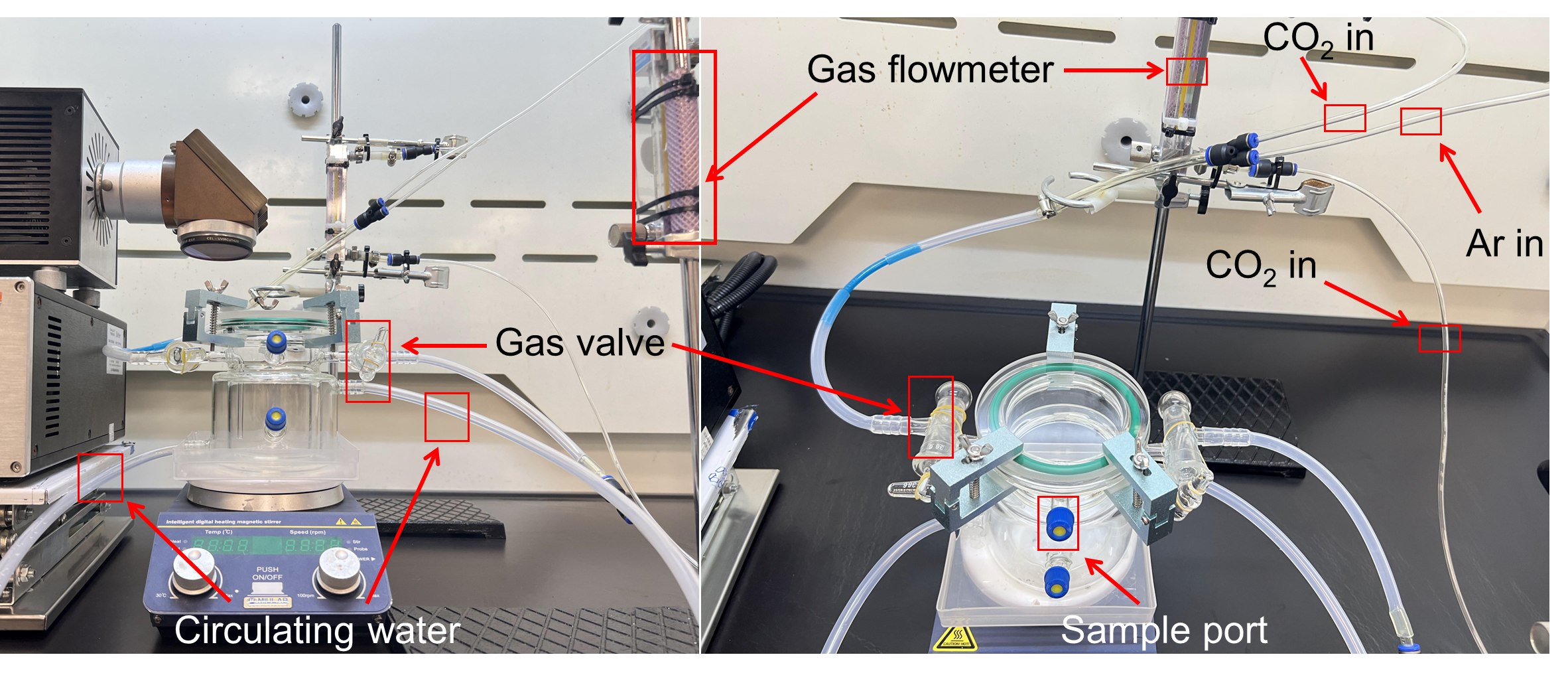


**Fig. S7** The photos for reaction device of photocatalytic CO_2_RR test.

**
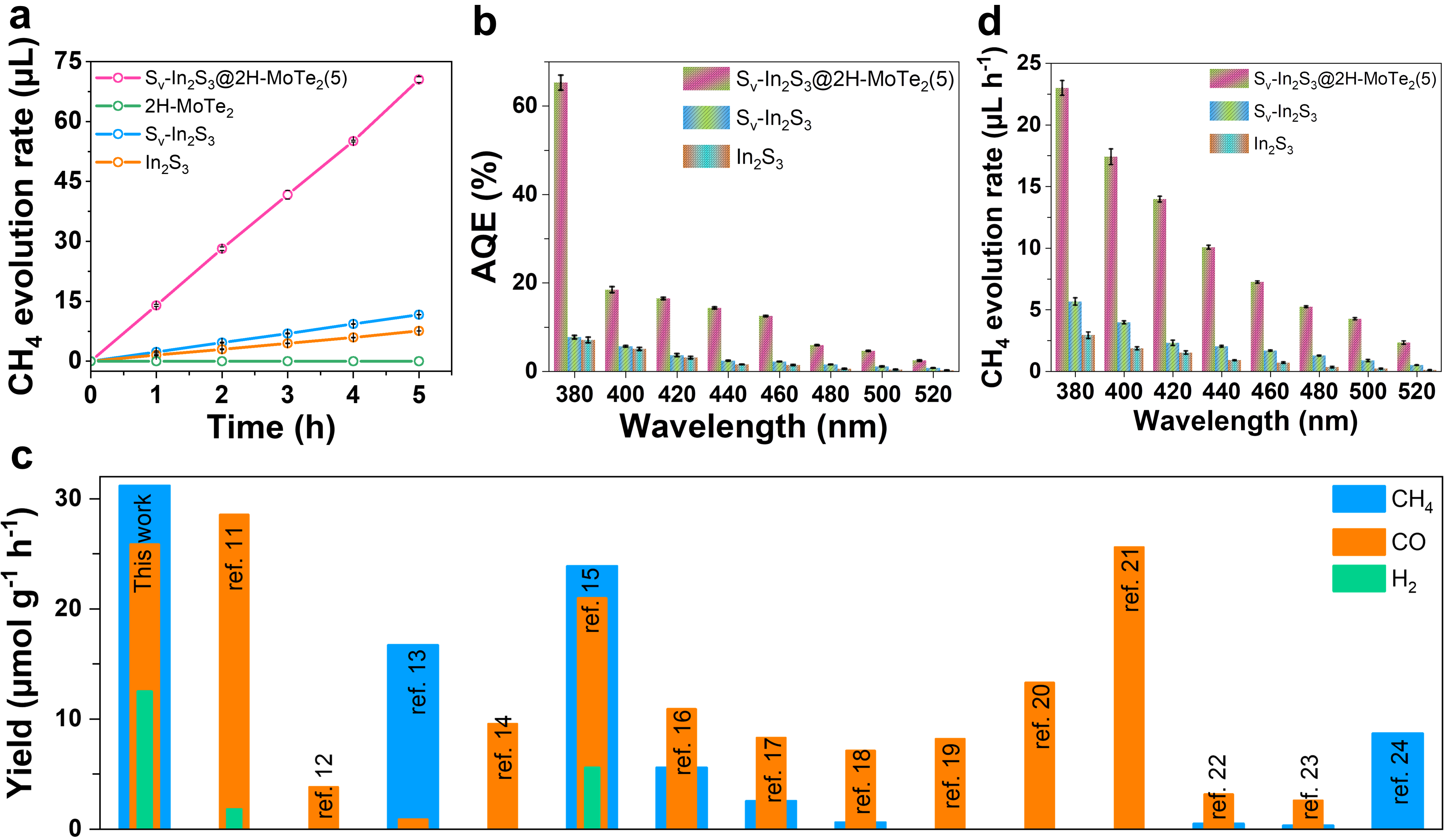
**

**Fig. S8 a** Photocatalytic CH_4_ evolution as a function of light irradiation time (KHCO_3_ solution). **b** AQE values of CO_2_ photoreduction to CH_4_ (KHCO_3_ solution). **c** Photocatalytic performance of S_v_-In_2_S_3_@2H-MoTe_2_(5) under monochromatic light irradiation (KHCO_3_ solution). **d** Comparison of photocatalytic CO_2_RR performance with visible-light irradiation [^[[11]](#endnote-12)^−,^[[12]](#endnote-13)^,^[[13]](#endnote-14)^,^[[14]](#endnote-15)^,^[[15]](#endnote-16)^,^[[16]](#endnote-17)^,^[[17]](#endnote-18)^,^[[18]](#endnote-19)^,^[[19]](#endnote-20)^,^[[20]](#endnote-21)^,^[[21]](#endnote-22)^,^[[22]](#endnote-23)^,^[[23]](#endnote-24)^,^[[24]](#endnote-25)^].


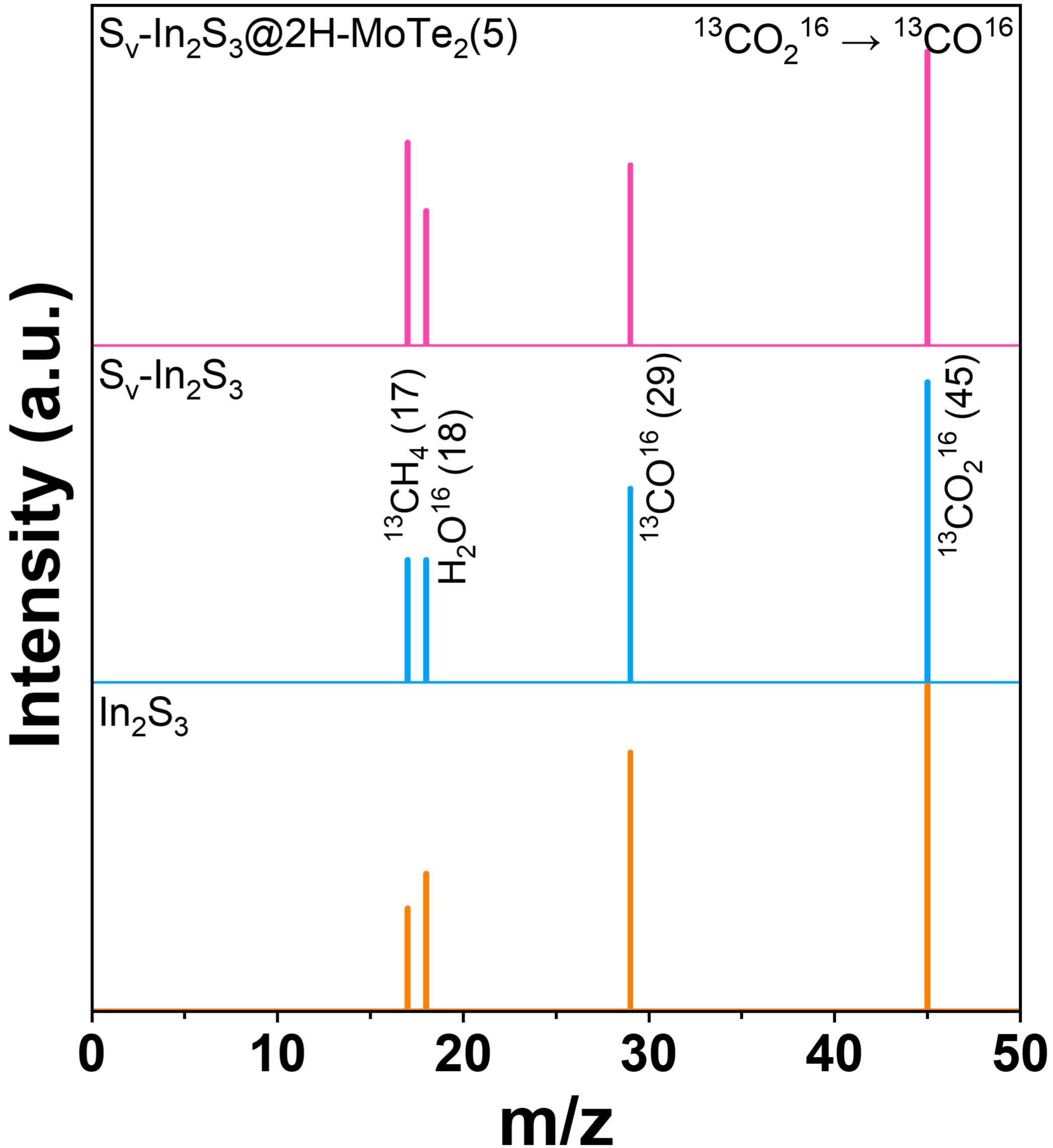


**Fig. S9** GC-MS analysis of product after ^13^CO_2_ photoreduction at *hν* = 14.3 eV with S_v_-In_2_S_3_@2H-MoTe_2_(5), S_v_-In_2_S_3_, and 2H-MoTe_2_, respectively (KHCO_3_ solution).


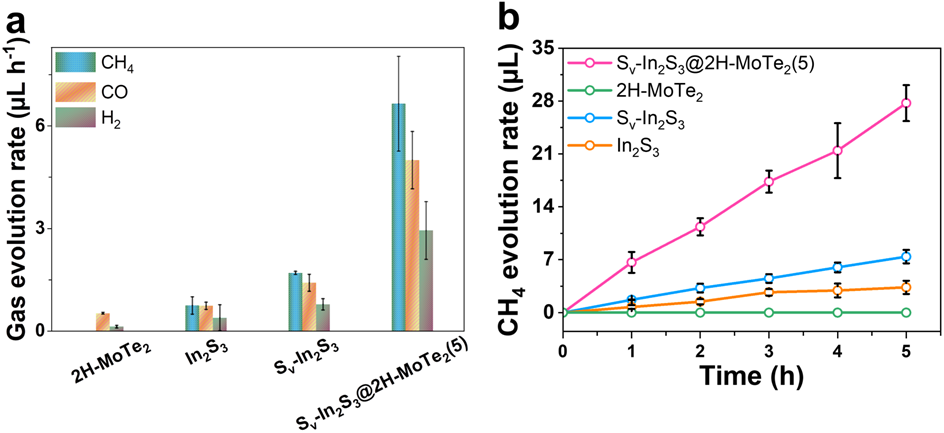


**Fig. S10** **a** Yields of CO, CH_4_, and H_2_ for photocatalysts (pure H_2_O solution). **b** Photocatalytic CH_4_ evolution as a function of light irradiation time (KHCO_3_ solution).


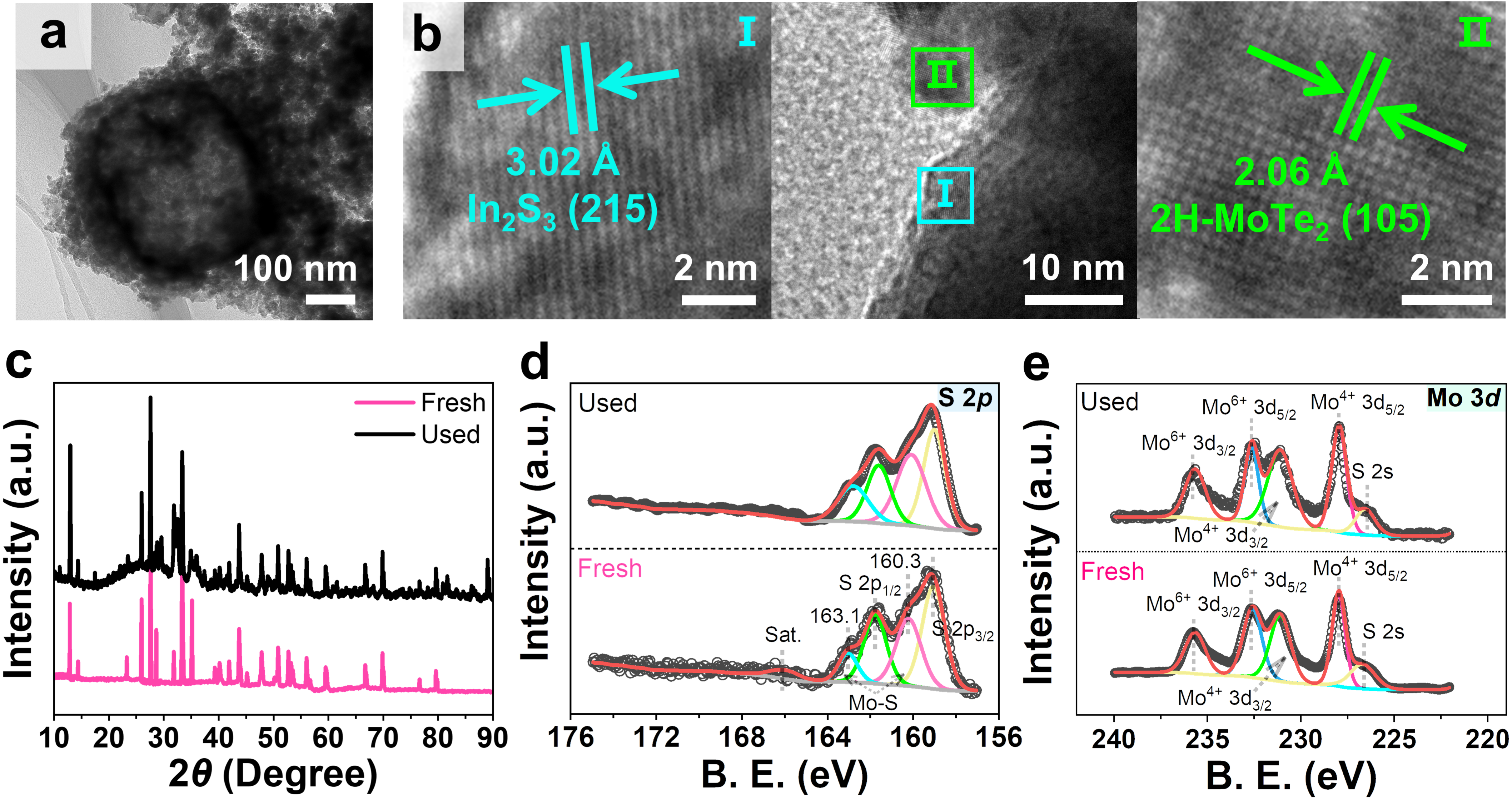


**Fig. S11 a** TEM and **b** HRTEM images of S_v_-In_2_S_3_@2H-MoTe_2_(5) after photocatalytic CO_2_RR (6 cycles). **c** XRD patterns of S_v_-In_2_S_3_@2H-MoTe_2_(5) after photocatalytic CO_2_RR (6 cycles), respectively. **d** S 2p and **e** Mo 3d XPS spectra of S_v_-In_2_S_3_@2H-MoTe_2_(5) after CO_2_RR test (6 cycles), respectively.


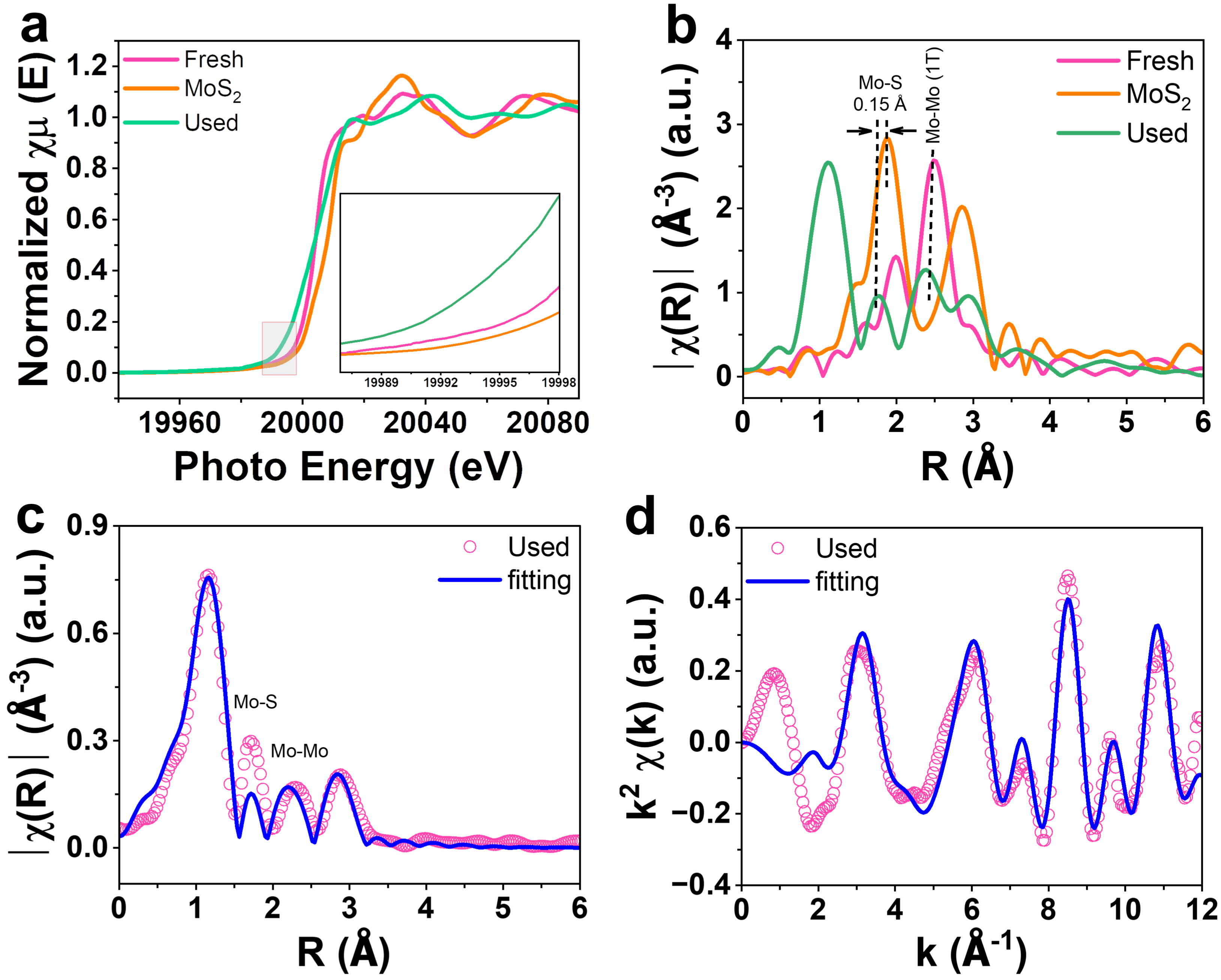


**Fig. S12 a** The comparison of Mo K-edge XANES spectra. **b** k^3^-weighted FT-EXAFS spectra of Mo at R space (the data of reference sample MoS_2_ come from Fig. 2c). **c** Mo K-edge EXAFS (points) and fit (line) for sample, shown in k^2^ weighted R-space after CO_2_RR test. **d** The comparison of Mo and In K-edge XANES spectra after photocatalytic CO_2_RR (6 cycles).


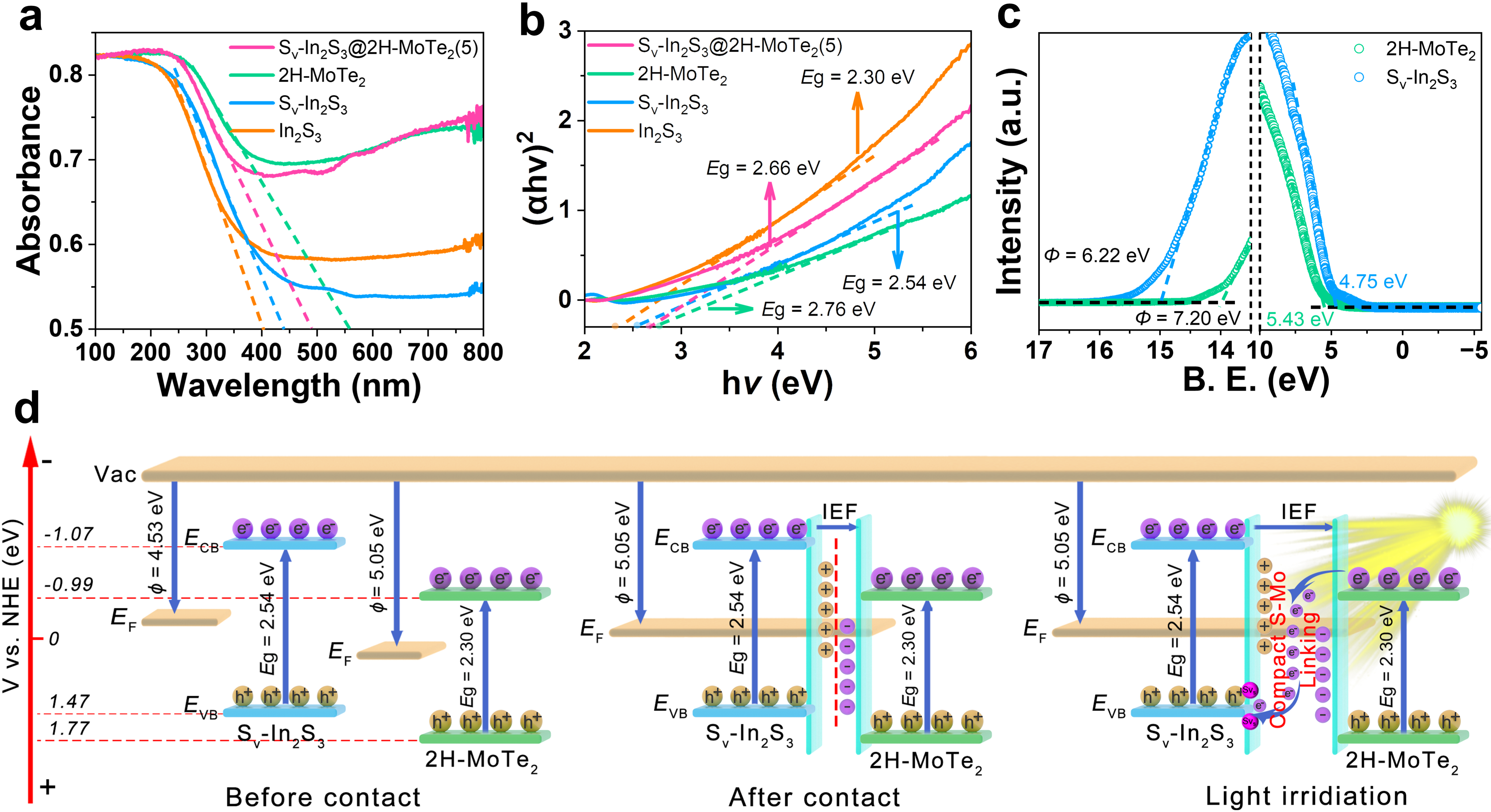


**Fig. S13 a** Ultraviolet-visible (UV-vis) diffuse reflectance spectra (DRS) and **b** (αhv)^2^ versus energy curves of samples. **c** Ultraviolet photoelectron spectroscopy (UPS) of S_v_-In_2_S_3_ and 2H-MoTe_2_, respectively. **d** Electronic structure and proposed charge transfer mechanism (“S”-scheme charge transfer mechanism).

As presented in Fig. S13a, S_v_-In_2_S_3_ and In_2_S_3_ mainly adsorb visible-light with adsorption edges at around 450 nm. After incorporation with 2H-MoTe_2_, the optical absorption curve of S_v_-In_2_S_3_@2H-MoTe_2_(5) shows a significant red shift, which is mainly owing to the strong light response of 2H-MoTe_2_ with an absorption edge of about 550 nm. Beyond that, the multiple light absorption effect produced by double shelled nanoboxes structure of S_v_-In_2_S_3_@2H-MoTe_2_(5) can effectively extend the light range and increase utilization of visible-light [^[[25]](#endnote-26)^,^[[26]](#endnote-27)^].

**
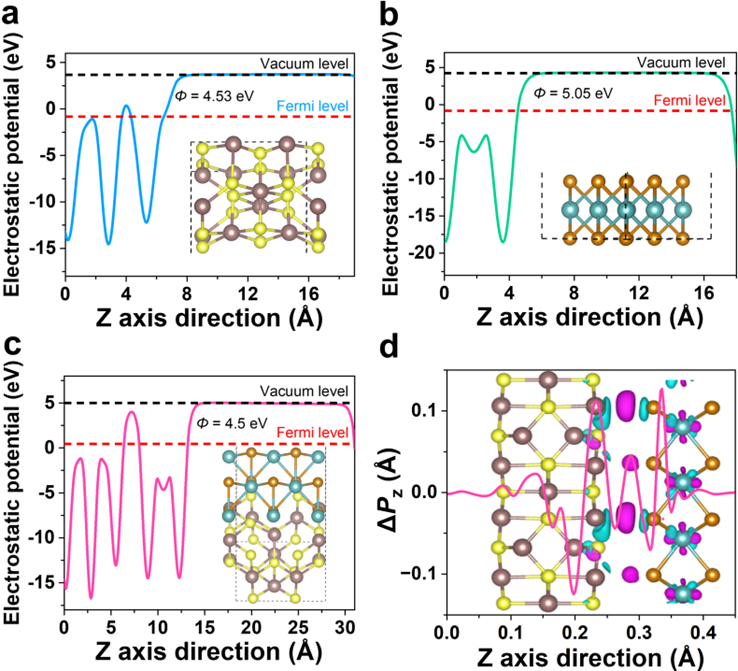
**

**Fig. S14** Electrostatic potential of **a** S_v_-In_2_S_3_, **b** 2H-MoTe_2_, and **c** S_v_-In_2_S_3_@2H-MoTe_2_(5), insets are models of In_2_S_3_, 2H-MoTe_2_, and S_v_-In_2_S_3_@2H-MoTe_2_(5), respectively. **d** Average charge density difference of S_v_-In_2_S_3_@2H-MoTe_2_(5) along Z axis direction (The magenta area (positive value) and cyan area (negative value) represent accumulation and consumption of electrons, respectively).

**Table S5** Work function value under vacuum environment and solvent effect.

| Slab | *E*_vac_ (eV) | *E*_F_ (eV) | *Φ* (eV) |
| --- | --- | --- | --- |
| 2H-MoTe_2_ | 4.26 | **−**0.79 | 5.05 |
| S_v_-In_2_S_3_ | 3.69 | **−**0.84 | 4.53 |
| S_v_-In_2_S_3_@2H-MoTe_2_(5) | 5.06 | 0.45 | 4.61 |

Due to different *E*_F_ and WF between S_v_-In_2_S_3_ and 2H-MoTe_2_, electrons in 2H-MoTe_2_ are transferred to S_v_-In_2_S_3_ as a way to equilibrate the *E*_F_ at the interface between two materials. After that, the photogenerated electrons on CB of 2H-MoTe_2_ are transferred to VB of S_v_-In_2_S_3_ by the induction of electrostatic potential and IEF effect, and then recombine with their photogenerated holes, namely “S”-scheme carrier mechanism [^[[27]](#endnote-28)^−,^[[28]](#endnote-29)^,^[[29]](#endnote-30)^].


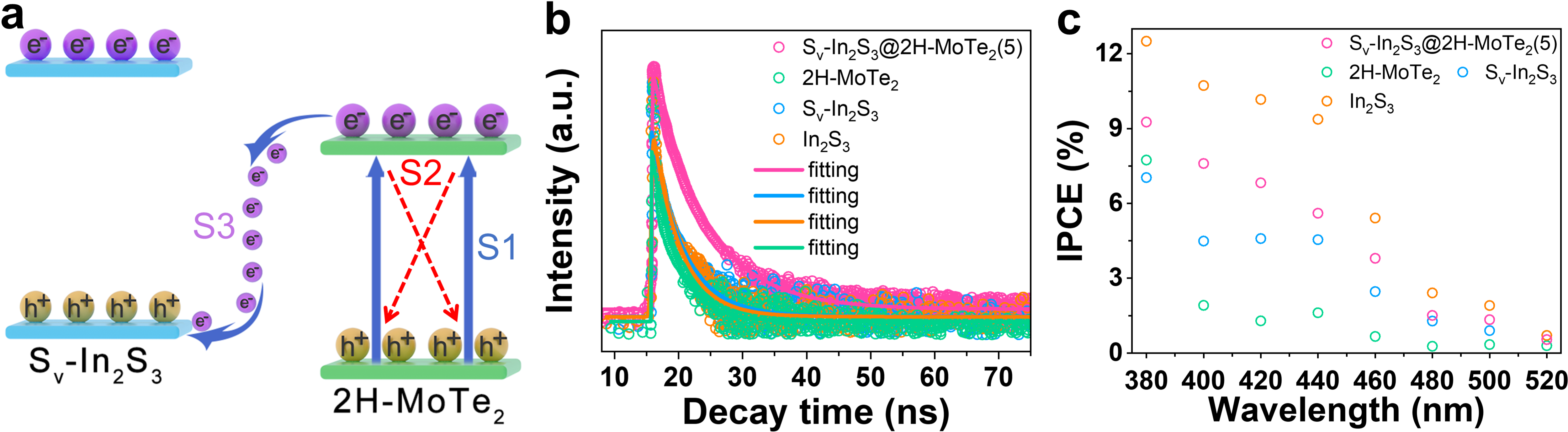


**Fig. S15 a** The excitons transport process of S_v_-In_2_S_3_@2H-MoTe_2_(5). **b** TRPL spectra and **c** IPCE profile (measured at an open circuit potential) of samples, respectively.

The exciton transport process of S_v_-In_2_S_3_@2H-MoTe_2_(5) can be divided into three parts as shown in Fig. S15a. S1 represents the exciton excited by high-energy photon. The competition between the Auger recombination process (S2) and interfacial charge transport process (S3) determine the lifetime of excitons and the density of photogenerated electrons and holes for participating photocatalytic overall water splitting reaction. The lifetime of excitons was evaluated through fs-TA spectra. The fast decay of fs-TA spectrum fitted via bi-exponential decay represents the nonradiative recombination (such as Auger process, insert in Fig. 4g) of samples. The S_v_-In_2_S_3_@2H-MoTe_2_(5) exhibits a longer excitons lifetime of 72.54 ps than S_v_-In_2_S_3_ (24.32 ps) and 2H-MoTe_2_ (7.23 ps), implying exciton extracting force by interfacial IEF (Fig. S13d) of S_v_-In_2_S_3_@2H-MoTe_2_(5) significantly suppresses the Auger recombination of 2H-MoTe_2_ (Fig. S15a).

**Table S6** The average fluorescence lifetime of samples.

| Samples | *τ*_1_(ns) | *A*_1_ (%) | *τ*_2_ (ns) | *A*_2_ (%) | Ave. τ(ns) |
| --- | --- | --- | --- | --- | --- |
| 2H-MoTe_2_ | 0.98 | 17 | 4.4 | 83 | 4.25 |
| In_2_S_3_ | 1.6 | 34 | 9.8 | 66 | 9.2 |
| S_v_-In_2_S_3_ | 11 | 22 | 15 | 78 | 14.3 |
| S_v_-In_2_S_3_@2H-MoTe_2_(5) | 12.3 | 28 | 22.6 | 72 | 20.8 |

* : *A*_1_+*A*_2_ =1

**Table S7** AQE and IQE_cr_ value of the S_v_-In_2_S_3_@2H-MoTe_2_(5), S_v_-In_2_S_3_, In_2_S_3_, and 2H-MoTe_2_ under different wavelength irradiation, respectively.

| Wavelength (nm) | S_v_-In_2_S_3_@2H-MoTe_2_(5) | | | S_v_-In_2_S_3_ | | | In_2_S_3_ | | | 2H-MoTe_2_ | | |
| --- | --- | --- | --- | --- | --- | --- | --- | --- | --- | --- | --- | --- |
|  | AQE (%) | Absorption | IQE_cr_ (%) | AQE (%) | Absorption | IQE_cr_ (%) | AQE (%) | Absorption | IQE_cr_ (%) | AQE (%) | Absorption | IQE_cr_ (%) |
| 380 | 65.29 | 0.6945 | 94.01 | 7.71 | 0.5985 | 12.88 | 7.07 | 0.6011 | 11.76 | 7.42 | 0.7060 | 10.51 |
| 400 | 18.45 | 0.6533 | 28.24 | 5.62 | 0.5819 | 9.66 | 5.05 | 0.5925 | 8.52 | 5.47 | 0.7008 | 7.81 |
| 420 | 16.46 | 0.6465 | 25.46 | 3.65 | 0.5691 | 6.41 | 3.09 | 0.5872 | 5.26 | 4.15 | 0.6947 | 5.97 |
| 440 | 14.3 | 0.6363 | 22.47 | 2.39 | 0.5580 | 4.28 | 1.54 | 0.5841 | 2.64 | 1.50 | 0.6941 | 2.16 |
| 460 | 12.48 | 0.6238 | 20 | 2.18 | 0.5527 | 3.94 | 1.40 | 0.5835 | 2.40 | 1.07 | 0.6952 | 1.54 |
| 480 | 5.91 | 0.6187 | 9.55 | 1.52 | 0.5489 | 2.77 | 0.54 | 0.5832 | 0.93 | 0.46 | 0.6967 | 0.66 |
| 500 | 4.6 | 0.6005 | 7.66 | 1.10 | 0.5469 | 2.01 | 0.38 | 0.5822 | 0.653 | 0.21 | 0.6979 | 0.30 |
| 520 | 2.42 | 0.5967 | 4.06 | 0.73 | 0.5445 | 1.34 | 0.22 | 0.5789 | 0.383 | 0.11 | 0.7021 | 0.16 |


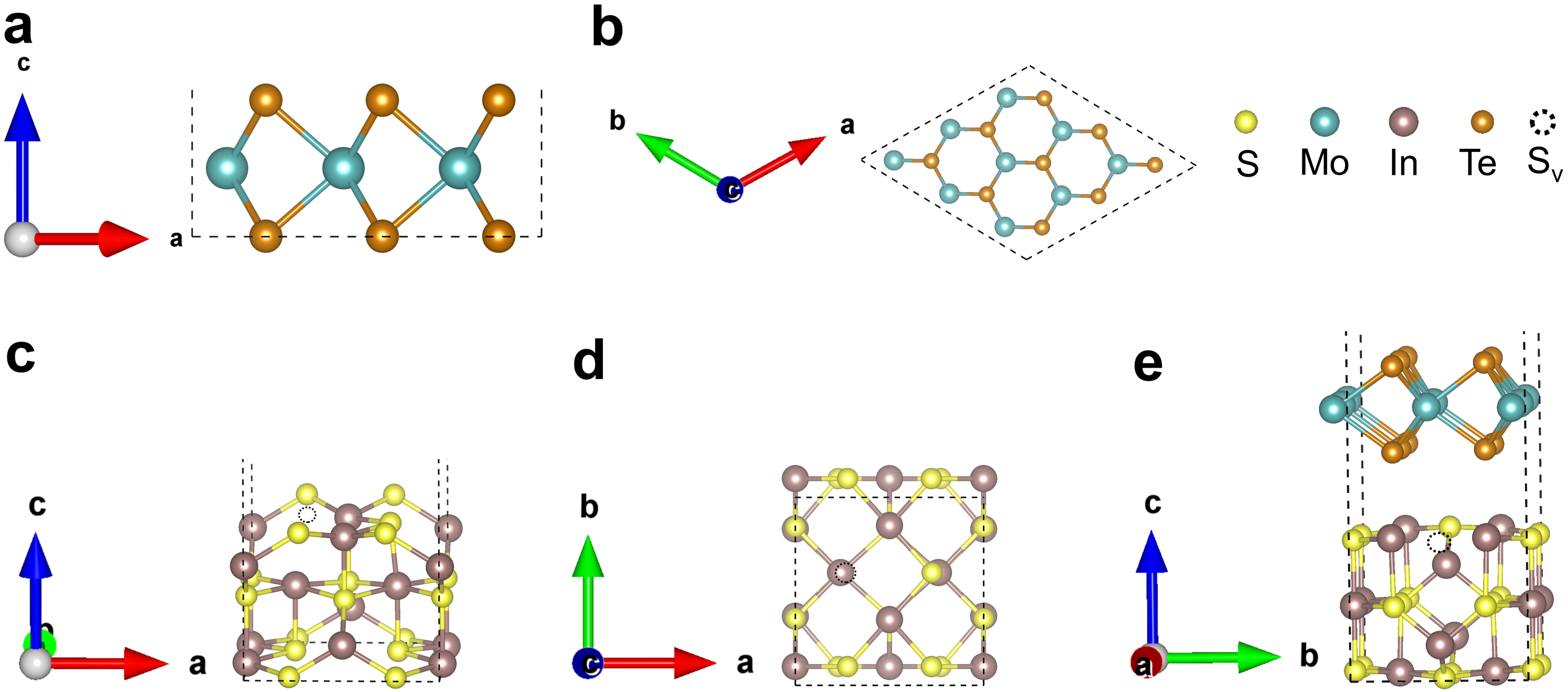


**Fig. S16** The optimized atomic models of **a,b** 2H-MoTe_2_, **c,d** S_v_-In_2_S_3_, and **e** S_v_-In_2_S_3_@2H-MoTe_2_(5) in theoretical calculations, respectively.


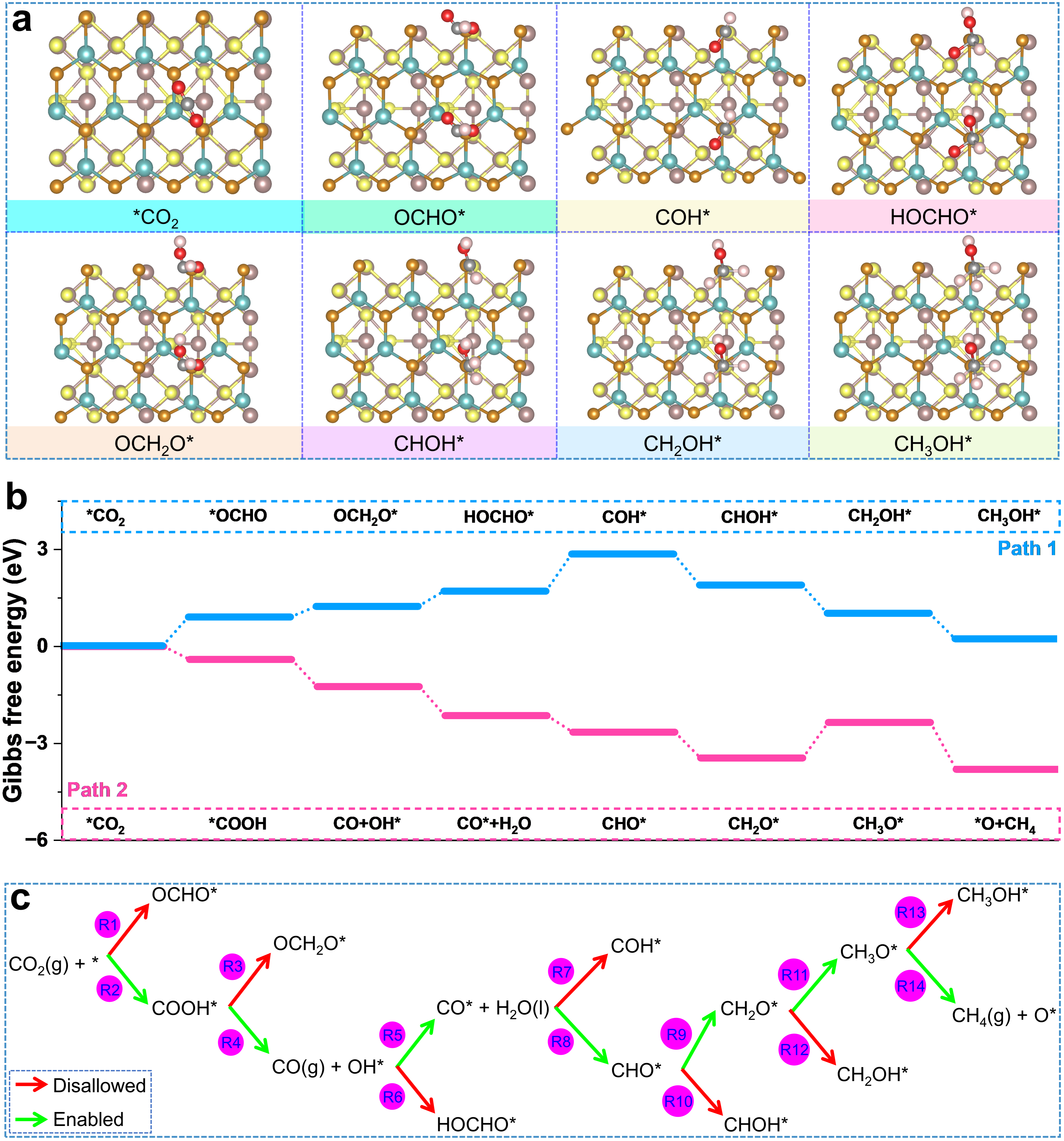


**Fig. S17 a** The schematic illustration of adsorption atomic structures during CO_2_RR process over S_v_-In_2_S_3_@2H-MoTe_2_(5) interfaces (Path 1). **b** Free energy diagrams for CO_2_ reduction to CH_4_ at 1.23 V bias over S_v_-In_2_S_3_@2H-MoTe_2_(5) interfaces with the maximum energy barrier (Path 1). **c** The search process for the minimum energy reaction pathways of CO_2_RR on S_v_-In_2_S_3_@2H-MoTe_2_(5) interfaces. The red arrows denote disallowed reaction paths, while green arrows stand for enabled ones.

All the CO_2_RR (R1 ~ R16) considered in the search process for the minimum energy reaction pathways of the CO_2_RR (Fig. S17c) can be written as:

| $CO_{2}(g)+H^{+}+e^{-}+\text{*}\to OCOH\text{*}$ | R1 |
| --- | --- |
| $CO_{2}(g)+H^{+}+e^{-}+\text{*}\to OCHO\text{*}$ | R2 |
| $OCHO\text{*}+H^{+}+e^{-}\to OCH_{2}O\text{*}$ | R3 |
| $OCHO\text{*}+H^{+}+e^{-}\to CO(g)+OH\text{*}$ | R4 |
| $OH\text{*}+H^{+}+e^{-}+CO(g)\to CO\text{*}+H_{2}O(\text{l})$ | R5 |
| $OH\text{*}+H^{+}+e^{-}+CO(g)\to HOCHO\text{*}$ | R6 |
| $CO\text{*}+H^{+}+e^{-}\to COH\text{*}$ | R7 |
| $CO\text{*}+H^{+}+e^{-}\to CHO\text{*}$ | R8 |
| $CHO\text{*}+H^{+}+e^{-}\to CH_{2}O\text{*}$ | R9 |
| $CHO\text{*}+H^{+}+e^{-}\to CHOH\text{*}$ | R10 |
| $CH_{2}O\text{*}+H^{+}+e^{-}\to CH_{3}O\text{*}$ | R11 |
| $CH_{2}O\text{*}+H^{+}+e^{-}\to CH_{2}OH\text{*}$ | R12 |
| $CH_{3}O\text{*}+H^{+}+e^{-}\to CH_{3}OH\text{*}$ | R13 |
| $CH_{3}O\text{*}+H^{+}+e^{-}\to CH_{4}(g)+O\text{*}$ | R14 |
| $O\text{*}+H_{2}O+H^{+}+e^{-}\to\text{*}OH$ | R15 |
| $\text{*}OH+H_{2}O+H^{+}+e^{-}\to H_{2}O$ | R16 |

The hydrogenation reactions (R17 ~ R18) for HER can be written as:

| $H^{+}+e^{-}+*\to H\text{*}$ | R17 |
| --- | --- |
| $H\text{*}+H^{+}+e^{-}\to H_{2}(\text{g})+*$ | R18 |

where * denotes the adsorption active site on the substrate.

We treated the charge of the system to refer to the widely-recognized computational hydrogen electrode (CHE) mode [10], in which each reaction step was regarded as a simultaneous transfer of the proton-electron pair as a function of the applied potential. Thus, free energy changes relative to an initial state of gaseous CO_2_ free above an empty surface can be rewritten as (U = 1.23 V and pH = 0) (R1 ~ R18):

| $\Delta G(OCOH*)= G(OCOH*) -[\frac{1}{2} G(H_{2})+G(*) +G(CO_{2})]$ | ES18 |
| --- | --- |
| $\Delta G(OCHO*)= G(OCHO*) -[\frac{1}{2} G(H_{2})+G(*) +G(CO_{2})]$ | ES19 |
| $\Delta G(OCH_{2}O*)= G(OCH_{2}O*) -[\frac{1}{2} G(H_{2})+G(*)]$ | ES20 |
| $\Delta G(CO\text{+}OH\text{*} )= G(CO)+ G(OH*)-[\frac{1}{2} G(H_{2})+G(OCHO*)]$ | ES21 |
| $\Delta G(CO\text{*+}H_{2}O )= G(CO*)+ G(H_{2}O)-[\frac{1}{2} G(H_{2})+G(OH*)]$ | ES22 |
| $\Delta G(HOCHO\text{*})= G(HOCHO*)-[\frac{1}{2} G(H_{2})+G(OH*)]$ | ES23 |
| $\Delta G(HOCHO\text{*})= G(HOCHO*)-[\frac{1}{2} G(H_{2})+G(OH*)]$ | ES24 |
| $\Delta G(COH\text{*})= G(COH*)-[\frac{1}{2} G(H_{2})+G(CO*)]$ | ES25 |
| $\Delta G(CH_{2}O*)= G(CH_{2}O*)-[\frac{1}{2} G(H_{2})+G(CHO*)]$ | ES26 |
| $\Delta G(CHOH*)= G(CHOH*)-[\frac{1}{2} G(H_{2})+G(CHO*)]$ | ES27 |
| $\Delta G(CH_{3}O*)= G(CH_{3}O*)-[\frac{1}{2} G(H_{2})+G(CH_{2}O*)]$ | ES28 |
| $\Delta G(CH_{2}OH\text{*})= G(CH_{2}OH\text{*} )-[\frac{1}{2} G(H_{2})+G(CH_{2}O*)]$ | ES29 |
| $\Delta G(CH_{3}OH\text{*})= G(CH_{3}OH\text{*} )-[\frac{1}{2} G(H_{2})+G(CH_{3}O*)]$ | ES30 |
| $\Delta G(CH_{4}\text{+}O\text{*} )= G(CH_{4})+G(O*)-[\frac{1}{2} G(H_{2})+G(CH_{3}O*)]$ | ES31 |
| $\Delta G(\text{*}OH )= G(\text{*}OH)-[\frac{1}{2} G(H_{2})+G(O*)+G(H_{2}O)]$ | ES32 |
| $\Delta G(H_{2}O )= G(H_{2}O)-[\frac{1}{2} G(H_{2})+G(OH*)+G(H_{2}O)]$ | ES33 |
| $\Delta G(H\text{*})= G(H*)-[\frac{1}{2} G(H_{2})+G(*)]$ | ES34 |
| $\Delta G(H_{2}\text{+}*)= G(H_{2})+G(*)-[\frac{1}{2} G(H_{2})+G(H*)]$ | ES35 |

The intermediates adsorption energy *E*_ads_ for H*, OH*, CO*, OCH_2_O*, CH_3_O*, COH*, and OCH_2_O*, etc. can be used as DFT ground state energy calculated as:

| $\Delta E(OCOH*)= E(OCOH*) -\frac{1}{2} E(H_{2})-E(*) -E(CO_{2})$ | ES36 |
| --- | --- |
| $\Delta E(OCHO*)= E(OCHO*) -\frac{1}{2} E(H_{2})-E(*) -E(CO_{2})$ | ES37 |
| $\Delta E(OCH_{2}O*)= E(OCH_{2}O*) -\frac{1}{2} E(H_{2})-E(*)$ | ES38 |
| $\Delta E(OH\text{*})= E(OH*)-\frac{1}{2} E(H_{2})-E(OCHO*)$ | ES39 |
| $\Delta E(CO*)= E(CO*)-\frac{1}{2} E(H_{2})-E(OH*)$ | ES40 |
| $\Delta E(HOCHO\text{*})= E(HOCHO*)-\frac{1}{2} E(H_{2})-E(OH*)$ | ES41 |
| $\Delta E(HOCHO\text{*})= E(HOCHO*)-\frac{1}{2} E(H_{2})-E(OH*)$ | ES42 |
| $\Delta E(COH\text{*})= E(COH*)-\frac{1}{2} E(H_{2})-E(CO*)$ | ES43 |
| $\Delta E(CH_{2}O\text{*})= E(CH_{2}O*)-\frac{1}{2} E(H_{2})-E(CHO*)$ | ES44 |
| $\Delta E(CHOH\text{*})= E(CHOH*)-\frac{1}{2} E(H_{2})-E(CHO*)$ | ES45 |
| $\Delta E(CH_{3}O*)= E(CH_{3}O*)-\frac{1}{2} E(H_{2})-E(CH_{2}O\text{*})$ | ES46 |
| $\Delta E(CH_{2}OH*)= E(CH_{2}OH\text{*} )-\frac{1}{2} E(H_{2})-E(CH_{2}O*)$ | ES47 |
| $\Delta E(CH_{3}OH\text{*})= E(CH_{3}OH\text{*} )-\frac{1}{2} E(H_{2})-E(CH_{3}O*)$ | ES48 |
| $\Delta E(O\text{*})= E(O*)-\frac{1}{2} E(H_{2})-E(CH_{3}O*)$ | ES49 |
| $\Delta E(H\text{*})= E(H*)-\frac{1}{2} E(H_{2})-E(*)$ | ES50 |

The evolution of CO_2_ to CO over S_v_-In_2_S_3_@2H-MoTe_2_(5) is a multistep proton-coupled electron transfer process as elucidated below:

| $CO_{2}\to CO_{2}*$ | ES51 |
| --- | --- |
| $CO_{2}\text{*}+H^{+}+e^{-}\to OCHO\text{*}$ | ES52 |
| $OCHO\text{*}+H^{+}+e^{-}\to CO(g)+OH\text{*}$ | ES53 |
| $OH\text{*}+H^{+}+e^{-}+CO(g)\to CO\text{*}+H_{2}O(\text{l})$ | ES54 |
| $CO\text{*}\to CO(g)$ | ES55 |


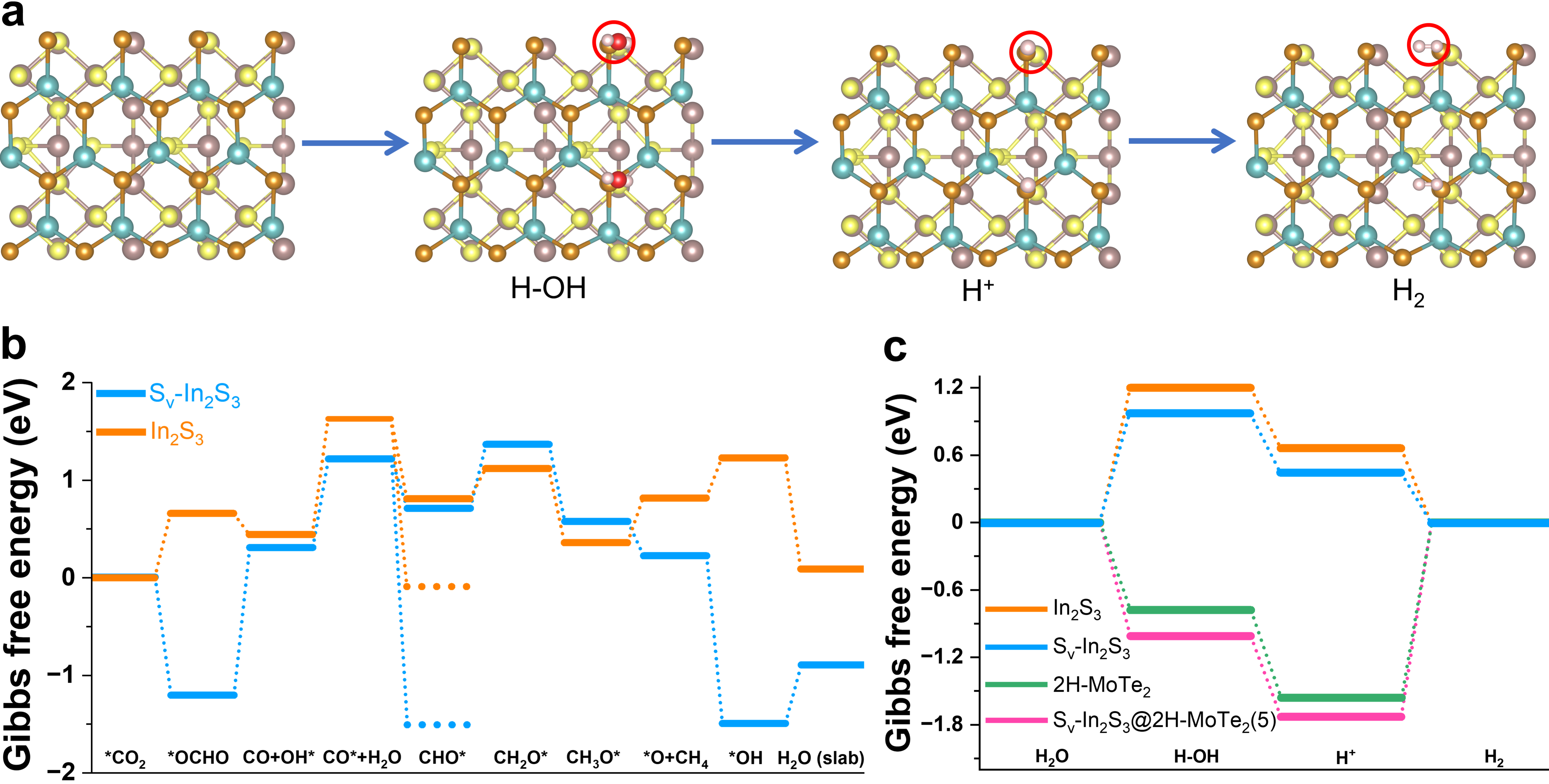


**Fig. S18** **a** The adsorption configuration of H_2_O* and H* intermediates on S_v_-In_2_S_3_@2H-MoTe_2_(5). The free energy diagrams for **b** CO_2_ reduction to CH_4_ at 1.23 V bias and **c** alkaline HER on over samples with the maximum energy barrier.

**Table S8** The Zero-pint energy correction (*E*_ZPE_), entropy contribution (*TS*, *T* = 298.15 K), and total energy (*E*_DFT_) of molecules in this work, are taken from the NIST database [9,^[[30]](#endnote-31)^].

| Species | *E*_DFT_ (eV) | *TS* (eV) | *T*∆*S* (eV) | *E*_ZPE_ (eV) | ∆*E*_ZPE_ (eV) | (∆*E*_ZPE_ − *T*∆*S*) (eV) |
| --- | --- | --- | --- | --- | --- | --- |
| CO_2_ (g) | **−**22.98 | 0.66 | / | 0.31 | / | / |
| CO (g) | **−**14.79 | 0.60 | / | 0.14 | / | / |
| CH_4_ (g) | **−**24.04 | 0.46 | / | 1.19 | / | / |
| H_2_O (l) [^[[31]](#endnote-32)^] | **−**14.21 | 0.67 | 0 | 0.59 | 0 | 0 |
| H_2_ (g) [^[[32]](#endnote-33)^,^[[33]](#endnote-34)^] | **−**6.76 | 0.42 | / | 0.27 | / | / |

**Table S9** Zero-pint energy correction (*E*_ZPE_), entropy contribution (*TS*, *T*=298.15 K), total energy (*E*_DFT_), and the Gibbs free energy (*G*) of molecules and adsorbates along the reaction pathway on S_v_-In_2_S_3_@2H-MoTe_2_(5), where * represents the adsorption site [9,30,^[[34]](#endnote-35)^,^[[35]](#endnote-36)^].

| Species | *TS* (eV) | *E*_ZPE_ (eV) |
| --- | --- | --- |
| OCH_2_O* | 0.21 | 0.92 |
| CH_2_O* | 0.17 | 0.74 |
| CH_3_O* | 0.25 | 1.04 |
| H* | 0.02 | 0.15 |
| O* | 0.07 | 0.07 |
| OH* | 0.08 | 0.33 |
| CO* | 0.26 | 0.15 |
| OCOH* | 0.24 | 0.62 |
| OCHO* | 0.17 | 0.42 |
| HOCH_2_O* | 0.24 | 1.24 |
| H_2_O* | 0.17 | 0.64 |

**Table S10** Summary of state-of-the-art photocatalytic CO_2_RR catalysts.

| Photocatalyst | Light Source | Experimental  Condition | Main Product | Yield  (µmol·g^−1^·h^−1^) | Reference |
| --- | --- | --- | --- | --- | --- |
| S_v_-In_2_S_3_@2H-MoTe_2_(5) | 300 W Xe lamp | Pure CO_2_ gas, gas-solid, water | CH_4_ | 31.18 | Our work |
|  |  |  | CO | 25.85 |  |
|  |  |  | H_2_ | 12.50 |  |
| Bi_2_O_2_(OH)(NO_3_) with surface Br ions | 300 W Xe lamp | Pure CO_2_ gas, gas-solid, water | CO | 8.12 | [^[[36]](#endnote-37)^] |
| Cs_2_SnI_6_ Perovskite Nanocrystal/SnS_2_ Nanosheet Heterojunction | A 150 mW·cm^−2^ Xe lamp (λ > 400 nm) | Pure CO_2_ gas, gas-solid, water, CH_3_OH | CH_4_ | 3.03 | [^[[37]](#endnote-38)^] |
| Single Cu Atom/Crystalline g-C_3_N_4_ | 300 W Xe lamp | Pure CO_2_ gas, gas-solid | CH_4_ | / | [^[[38]](#endnote-39)^] |
|  |  |  | CO | 3.09 |  |
| Monolayer N-doped graphene on CdS hollow spheres | A 350 W Xe lamp (λ > 420 nm) | Pure CO_2_ gas, gas-solid, water | CH_4_ | 0.33 | [23] |
|  |  |  | CO | 2.59 |  |
| UiO-66/carbon nitride nanosheet heterogeneous photocatalyst | A 300 W Xe lamp (800 nm >λ > 400 nm) | Pure CO_2_ gas, gas-solid, water | CO | 9.90 | [^[[39]](#endnote-40)^] |
| Perovskite-like PbBiO_2_Br | 300 W Xe lamp | Pure CO_2_ gas, gas-solid | CO | 1.24 | [^[[40]](#endnote-41)^] |
| ICN-16 | 300 W Xe lamp | Pure CO_2_ gas | CO | 398.87 | [^[[41]](#endnote-42)^] |
| GGS/GS | 300 W Xe lamp | Pure CO_2_ gas, gas-solid, water | C_2_H_4_ | 335.67 | [^[[42]](#endnote-43)^] |
| ZnAl-LDH nanosheets | 300 W Xe lamp | Pure CO_2_ gas, gas-solid | CO | 7.60 | [^[[43]](#endnote-44)^] |
| Cs_3_Bi_2_I_9_ perovskite nanocrystals | 32 W UV lamp | Pure CO_2_ gas, gas-solid, water | CH_3_OH | 1.49 | [^[[44]](#endnote-45)^] |
|  |  |  | CO | 7.76 |  |
| Atomically-thin Bi_2_MoO_6_ nanosheets with vacancy pairs | 300 W Xe lamp | Pure CO_2_ gas, gas-solid | CO | 3.62 | [^[[45]](#endnote-46)^] |
| Ultrathin Pb_0.6_Bi_1.4_O_2_Cl_2_ | 300 W Xe lamp | Air, gas-solid | CO | 5.16 | [^[[46]](#endnote-47)^] |
|  |  |  | CH_4_ | 0.62 |  |
|  |  |  | CH_3_OH | 1.98 |  |

**References**

1. [] J. Nai, S. Wang, Y. Bai, L. Guo, Amorphous Ni(OH)_2_ Nanoboxes: Fast Fabrication and Enhanced Sensing for Glucose, Small **9**, 3147–3152 (2013). <https://doi.org/10.1002/smll.201203076> [↑](#endnote-ref-2)
2. [] Y. Fang, B.Y. Guan, D. Luan, X.W. Lou, Synthesis of CuS@CoS_2_ double-shelled nanoboxes with enhanced sodium storage properties, Angew. Chem. Int. Ed. **58**, 7739–7743 (2019). <https://doi.org/10.1002/anie.201902583> [↑](#endnote-ref-3)
3. [] B. Ravel, M. Newville, ATHENA, ARTEMIS, HEPHAESTUS: data analysis for X-ray absorption spectroscopy using IFEFFIT, J. Synchrotron Rad. **12**, 537–541 (2005). <https://doi.org/10.1107/S0909049505012719> [↑](#endnote-ref-4)
4. [] H. Funke, A.C. Scheinost, M. Chukalina, Wavelet analysis of extended x-ray absorption fine structure data, Phys. Rev. B **71**, 094110 (2005). <https://doi.org/10.1103/PhysRevB.71.094110> [↑](#endnote-ref-5)
5. [] H. Funke, M. Chukalina, A.C. Scheinost, A new FEFF-based wavelet for EXAFS data analysis, J. Synchrotron Rad. **14**, 426–432 (2007). <https://doi.org/10.1107/S0909049507031901> [↑](#endnote-ref-6)
6. [] C. Cheng, B. He, J. Fan, B. Cheng, S. Cao et al., An Inorganic/Organic S-Scheme Heterojunction H_2_-Production Photocatalyst and its Charge Transfer Mechanism, Adv. Mater. **33**, 2100317 (2021). <https://doi.org/10.1002/adma.202100317> [↑](#endnote-ref-7)
7. [] M. Ahmad, X. Quan, S. Chen, H. Yu, Z. Zeng, Operating redox couple transport mechanism for enhancing photocatalytic H_2_ generation of Pt and CrO_x_-decorated ZnCdS nanocrystals, Appl. Catal. B Environ. **283**, 119601 (2021). <https://doi.org/10.1016/j.apcatb.2020.119601> [↑](#endnote-ref-8)
8. [] A. Zhang, Y. Liang, H. Li, X. Zhao, Y. Chen et al., Harmonizing the Electronic Structures of the Adsorbate and Catalysts for Efficient CO_2_ Reduction, Nano Lett. **9**, 6547–6553 (2019). <https://doi.org/10.1021/acs.nanolett.9b02782> [↑](#endnote-ref-9)
9. [] Z. Wang, J. Zhu, X. Zu, Y. Wu, S. Shang et al., Selective CO_2_ Photoreduction to CH_4_ via P^dᵟ+^-assisted Hydrodeoxygenation over CeO_2_ Nanosheets, Angew. Chem. Int. Ed. **61**, e202203249 (2022). <https://doi.org/10.1002/anie.202203249> [↑](#endnote-ref-10)
10. [] L. Ju, X. Tan, X. Mao, Y. Gu, S. Smith et al., Controllable CO_2_ electrocatalytic reduction via ferroelectric switching on single atom anchored In_2_Se_3_ monolayer, Nat. Commun. **12**, 5128 (2021). <https://doi.org/10.1038/s41467-021-25426-5> [↑](#endnote-ref-11)
11. [] B. Yu, Y. Wu, F. Meng, Q. Wang, X. Jia et al., Formation of hierarchical Bi_2_MoO_6_/ln_2_S_3_ S-scheme heterojunction with rich oxygen vacancies for boosting photocatalytic CO_2_ reduction, Chem. Eng. J. **429**, 132456 (2022). <https://doi.org/10.1016/j.cej.2021.132456> [↑](#endnote-ref-12)
12. [] W. Dong, J. Jia, Y. Wang, J. An, O.Y. Yang et al., Visible-light-driven solvent-free photocatalytic CO_2_ reduction to CO by Co-MOF/Cu_2_O heterojunction with superior selectivity, Chem. Eng. J. **438**, 135622 (2022). <https://doi.org/10.1016/j.cej.2022.135622> [↑](#endnote-ref-13)
13. [] J. Jiang, X. Wang, Q. Xu, Z. Mei, L. Duan et al., Understanding dual-vacancy heterojunction for boosting photocatalytic CO_2_ reduction with highly selective conversion to CH_4_, Appl. Catal. B Environ. **316**, 121679 (2022). <https://doi.org/10.1016/j.apcatb.2022.121679> [↑](#endnote-ref-14)
14. [] Z. Zhang, M. Wang, Z. Chi, W. Li, H. Yu et al., Internal electric field engineering step-scheme-based heterojunction using lead-free Cs_3_Bi_2_Br_9_ perovskite-modified In_4_SnS_8_ for selective photocatalytic CO_2_ reduction to CO, Appl. Catal. B Environ. **313**, 121426 (2022). <https://doi.org/10.1016/j.apcatb.2022.121426> [↑](#endnote-ref-15)
15. [] Y. Chai, Y. Chen, J. Shen, M. Ni, B. Wang et al., Distortion of the Coordination Structure and High Symmetry of the Crystal Structure in In_4_SnS_8_ Microflowers for Enhancing Visible-Light Photocatalytic CO_2_ Reduction, ACS Catal. **11**, 11029−11039 (2021). <https://doi.org/10.1021/acscatal.1c02937> [↑](#endnote-ref-16)
16. [] W. Gao, S. Li, H. He, X. Li, Z. Cheng et al., Vacancy-defect modulated pathway of photoreduction of CO_2_ on single atomically thin AgInP_2_S_6_ sheets into olefiant gas, Nat. Commun. **12**, 4747 (2021). <https://doi.org/10.1038/s41467-021-25068-7> [↑](#endnote-ref-17)
17. [] J. Jiang, X. Zou, Z. Mei, S. Cai, Q. An et al., Understanding rich oxygen vacant hollow CeO_2_@MoSe_2_ heterojunction for accelerating photocatalytic CO_2_ reduction, J. Colloid Interf. Sci. **611**, 644–653 (2022). <https://doi.org/10.1016/j.jcis.2021.12.108> [↑](#endnote-ref-18)
18. [] Y. Liu, D. Shen, Q. Zhang, Y. Lin, F. Peng, Enhanced photocatalytic CO_2_ reduction in H_2_O vapor by atomically thin Bi_2_WO_6_ nanosheets with hydrophobic and nonpolar surface, Appl. Catal. B Environ. **283**, 119630 (2021). <https://doi.org/10.1016/j.apcatb.2020.119630> [↑](#endnote-ref-19)
19. [] Y. Huang, K. Wang, T. Guo, J. Li, X. Wu et al., Construction of 2D/2D Bi_2_Se_3_/g-C_3_N_4_ nanocomposite with High interfacial charge separation and photo-heat conversion efficiency for selective photocatalytic CO_2_ reduction, Appl. Catal. B Environ. **277**, 119232 (2020). <https://doi.org/10.1016/j.apcatb.2020.119232> [↑](#endnote-ref-20)
20. [] X. Wang, Y. Wang, M. Gao, J. Shen, X. Pu et al., BiVO_4_/Bi_4_Ti_3_O_12_ heterojunction enabling efficient photocatalytic reduction of CO_2_ with H_2_O to CH_3_OH and CO, Appl. Catal. B Environ. **270**, 118876 (2020). <https://doi.org/10.1016/j.apcatb.2020.118876> [↑](#endnote-ref-21)
21. [] X. Chen, Q. Li, J. Li, J. Chen, H. Jia, Modulating charge separation via in situ hydrothermal assembly of low content Bi_2_S_3_ into UiO-66 for efficient photothermocatalytic CO_2_ reduction, Appl. Catal. B Environ. **270**, 118915 (2020). <https://doi.org/10.1016/j.apcatb.2020.118915> [↑](#endnote-ref-22)
22. [] X. Jin, C. Lv, X. Zhou, H. Xie, S. Sun et al., A bismuth rich hollow Bi_4_O_5_Br_2_ photocatalyst enables dramatic CO_2_ reduction activity, Nano Energy **64**, 103955 (2019). <https://doi.org/10.1016/j.nanoen.2019.103955> [↑](#endnote-ref-23)
23. [] C. Bie, B. Zhu, F. Xu, L. Zhang, J. Yu, In Situ Grown Monolayer N-Doped Graphene on CdS Hollow Spheres with Seamless Contact for Photocatalytic CO_2_ Reduction, Adv. Mater. **31**, 1902868 (2019). <https://doi.org/10.1002/adma.201902868> [↑](#endnote-ref-24)
24. [] X. Li, Y. Sun, J. Xu, Y. Shao, J. Wu et al., Selective visible-light-driven photocatalytic CO_2_ reduction to CH_4_ mediated by atomically thin CuIn_5_S_8_ layers, Nat. Energy **4**, 690–699 (2019). <https://doi.org/10.1038/s41560-019-0431-1> [↑](#endnote-ref-25)
25. [] P. Zhang, D. Luan, X.W. (David) Lou, Fabrication of CdS Frame-in-Cage Particles for Efficient Photocatalytic Hydrogen Generation under Visible-Light Irradiation, Adv. Mater. **32**, 2004561 (2020). <https://doi.org/10.1002/adma.202004561> [↑](#endnote-ref-26)
26. [] K. Li, Y. Cai, X. Yang, S. Wang, C. Teng et al., H_2_S Involved Photocatalytic System: A Novel Syngas Production Strategy by Boosting the Photoreduction of CO_2_ While Recovering Hydrogen from the Environmental Toxicant, Adv. Funct. Mater. **32**, 2113002 (2022). <https://doi.org/10.1002/adfm.202113002> [↑](#endnote-ref-27)
27. [] S. Mukherjee, D. Dutta, M. Uzhansky, E. Koren, Excitons and light-emission in semiconducting MoSi_2_X_4_ two-dimensional materials, npj 2D Mater Appl. **6**, 37 (2022). <https://doi.org/10.1038/s41699-022-00355-z> [↑](#endnote-ref-28)
28. [] Y. Geng, D. Chen, N. Li, Q. Xu, H. Li et al., Z-Scheme 2D/2D α-Fe_2_O_3_/g-C_3_N_4_ heterojunction for photocatalytic oxidation of nitric oxide, Appl. Catal. B Environ. **280**, 119409 (2021). <https://doi.org/10.1016/j.apcatb.2020.119409> [↑](#endnote-ref-29)
29. [] Z. Zhang, M. Wang, Z. Chi, W. Li, H. Yu et al., Internal electric field engineering step-scheme-based heterojunction using lead-free Cs_3_Bi_2_Br_9_ perovskite-modified In_4_SnS_8_ for selective photocatalytic CO_2_ reduction to CO, Appl. Catal. B Environ. **313**, 121426 (2022). <https://doi.org/10.1016/j.apcatb.2022.121426> [↑](#endnote-ref-30)
30. [] WebBook NC. <07838353.pdf>. <https://webbooknistgov/chemistry/> [↑](#endnote-ref-31)
31. [] S. Fang, X. Zhu, X. Liu, J. Gu, W. Liu et al., Uncovering near-free platinum single-atom dynamics during electrochemical hydrogen evolution reaction, Nat. Commun. **11**, 1029 (2020). <https://doi.org/10.1038/s41467-020-14848-2> [↑](#endnote-ref-32)
32. [] NIST-JANAF Thermochemical Tables. <https://janaf.nist.gov/> [↑](#endnote-ref-33)
33. [] J. Rossmeisl, A. Logadottir, J. K. Nørskov, Electrolysis of water on (oxidized) metal surfaces, Chem. Phys. **319**, 178–184 (2005). <https://doi.org/10.1016/j.chemphys.2005.05.038> [↑](#endnote-ref-34)
34. [] D. Raciti, L. Cao, K.J.T. Livi, P.F. Rottmann, X. Tang et al., Low-overpotential electroreduction of carbon monoxide using copper nanowires, ACS Catal. **7**, 4467–4472 (2017). <https://doi.org/10.1021/acscatal.7b01124> [↑](#endnote-ref-35)
35. [] A.A. Peterson, F. Abild-Pedersen, F. Studt, J. Rossmeisl, J.K. Norskov, How copper catalyzes the electroreduction of carbon dioxide into hydrocarbon fuels, Energ. Environ. Sci. **3**, 1311–1315 (2010). <https://doi.org/10.1039/C0EE00071J> [↑](#endnote-ref-36)
36. [] L. Hao, L. Kang, H. Huang, L. Ye, K. Han et al., Surface-Halogenation-Induced Atomic-Site Activation and Local Charge Separation for Superb CO_2_ Photoreduction, Adv. Mater. **31**, 1900546 (2019). <https://doi.org/10.1002/adma.201900546> [↑](#endnote-ref-37)
37. [] X. Wang, Y. Huang, J. Liao, Y. Jiang, L. Zhou et al., In Situ Construction of a Cs_2_SnI_6_ Perovskite Nanocrystal/SnS_2_ Nanosheet Heterojunction with Boosted Interfacial Charge Transfer, J. Am. Chem. Soc. **141**, 13434–13441 (2019). <https://doi.org/10.1021/jacs.9b04482> [↑](#endnote-ref-38)
38. [] J. Wu, X. Li, W. Shi, P. Ling, Y. Sun et al., Efficient Visible-Light-Driven CO_2_ Reduction Mediated by Defect-Engineered BiOBr Atomic Layers, Angew. Chem. Int. Ed. **57**, 8719–8723 (2018). <https://doi.org/10.1002/ange.201803514> [↑](#endnote-ref-39)
39. [] L. Shi, T. Wang, H. Zhang, K. Chang, J. Ye, Electrostatic Self-Assembly of Nanosized Carbon Nitride Nanosheet onto a Zirconium Metal-Organic Framework for Enhanced Photocatalytic CO_2_ Reduction, Adv. Funct. Mater. **25**, 5360–5367 (2015). <https://doi.org/10.1002/adfm.201502253> [↑](#endnote-ref-40)
40. [] B. Wang, J. Di, L. Lu, S. Yan, G. Liu et al., Sacrificing ionic liquid-assisted anchoring of carbonized polymer dots on perovskite-like PbBiO_2_Br for robust CO_2_ photoreduction, Appl. Catal. B Environ. **254**, 551–559 (2019). <https://doi.org/10.1016/j.apcatb.2019.04.068> [↑](#endnote-ref-41)
41. [] C. Ding, X. Lu, B. Tao, L. Yang, X. Xu et al., Interlayer Spacing Regulation by Single-Atom Indium^𝜹+^–N_4_ on Carbon Nitride for Boosting CO_2_/CO Photo-Conversion, Adv. Funct. Mater. 2302824 (2023). <https://doi.org/10.1002/adfm.202302824> [↑](#endnote-ref-42)
42. [] J. Wang, C. Yang, L. Mao, X. Cai, Z. Geng et al., Regulating the Metallic Cu-Ga Bond by S Vacancy for Improved Photocatalytic CO_2_ Reduction to C_2_H_4_, Adv. Funct. Mater. **33**, 2213901 (2023). <https://doi.org/10.1002/adfm.202213901> [↑](#endnote-ref-43)
43. [] Y. Zhao, G. Chen, T. Bian, C. Zhou, G. I. N. Waterhouse et al., Defect-Rich Ultrathin ZnAl-Layered Double Hydroxide Nanosheets for Efficient Photoreduction of CO_2_ to CO with Water, Adv. Mater. **27**, 7824–7831 (2015). <https://doi.org/10.1002/adma.201503730> [↑](#endnote-ref-44)
44. [] S. S. Bhosale, A. K. Kharade, E. Jokar, A. Fathi, S. Chang et al., Mechanism of Photocatalytic CO_2_ Reduction by Bismuth-Based Perovskite Nanocrystals at the Gas-Solid Interface, J. Am. Chem. Soc. **141**, 20434–20442 (2019). <https://doi.org/10.1021/jacs.9b11089> [↑](#endnote-ref-45)
45. [] J. Di, X. Zhao, C. Lian, M. Ji, J. Xia et al., Atomically-thin Bi_2_MoO_6_ nanosheets with vacancy pairs for improved photocatalytic CO_2_ reduction, Nano Energy **61**, 54–59 (2019). <https://doi.org/10.1016/j.nanoen.2019.04.029> [↑](#endnote-ref-46)
46. [] X. Feng, R. Zheng, C. Gao, W. Wei, J. Peng et al., Unlocking bimetallic active sites via a desalination strategy for photocatalytic reduction of atmospheric carbon dioxide, Nat. Commun. **13**, 2146 (2022). <https://doi.org/10.1038/s41467-022-29671-0> [↑](#endnote-ref-47)
